# ddOTs: A multiplexed quantitative ddPCR approach for resolving overlapping hepatitis B virus transcripts to decipher cccDNA-driven transcription

**DOI:** 10.1371/journal.ppat.1014464

**Published:** 2026-07-30

**Authors:** Nazim Sarica, Oceane Lopez, João Diogo Dias, Victor Boschetti, Irene Meki, Basile Jay, Gael Petitjean, David Durantel, Christine Neuveut

**Affiliations:** 1 Institut de Génétique Humaine. Laboratoire de Virologie Moléculaire, Université de Montpellier, CNRS, Montpellier, France; 2 Centre International de Recherche en Infectiologie (CIRI), INSERM, U1111, Université Claude Bernard Lyon 1, CNRS, UMR5308, ENS de Lyon, France; 3 Institut de Génétique Humaine. Laboratoire de Virologie Moléculaire, Université de Montpellier, CNRS, INSERM, Montpellier, France; Pennsylvania State University College of Medicine: Penn State College of Medicine, UNITED STATES OF AMERICA

## Abstract

Chronic hepatitis B virus infection persists as a global health crisis, driving life-threatening liver pathologies such as hepatocellular carcinoma. Central to HBV’s resilience is the covalently closed circular DNA (cccDNA), a viral minichromosome that orchestrates viral transcription and sustains infection. Dissecting cccDNA-driven transcription remains a formidable challenge due to the dense overlap of viral open reading frames, which generates RNA transcripts with shared sequences, complicating precise quantification and hindering efforts to unravel HBV’s transcriptional regulation. To address this bottleneck, we developed a multiplexed assay named ddPCR for Overlapping Transcripts (ddOTs) capable of simultaneously quantifying all major HBV RNA species, including splice variants, with unprecedented specificity and sensitivity. This method overcomes current limitations by leveraging the high-resolution power of ddPCR to deconvolute overlapping transcripts, enabling direct interrogation of individual promoter/enhancer activities and RNA stability. By offering a cost-effective, scalable solution for precise RNA profiling in rare biological samples, this breakthrough tool unlocks new avenues for exploring cccDNA biology and accelerating antiviral drug development.

## Author summary

Chronic hepatitis B virus infection is a leading cause of liver disease and cancer worldwide. The virus persists in infected liver cells through a stable DNA molecule called covalently closed circular DNA, which acts as a template for viral gene expression. Understanding how this DNA produces the different viral RNA molecules is essential to develop new curative treatments. However, this has been difficult because the virus has a compact genome with overlapping genes, making its RNA transcripts hard to distinguish using standard techniques. In our study, we developed a new digital droplet–based approach that allows precise and simultaneous measurement of all hepatitis B virus RNA species. This method allows accurate and detailed quantification of viral RNA transcripts, providing a useful additional tool to examine aspects of viral gene expression. By enabling such analyses even from rare or small biological samples, our approach can facilitate future studies aimed at understanding viral persistence and identifying potential antiviral targets. Finally, this technique could also be adapted to study other organisms whose genomes contain overlapping genes.

## Introduction

**Fig 1:**
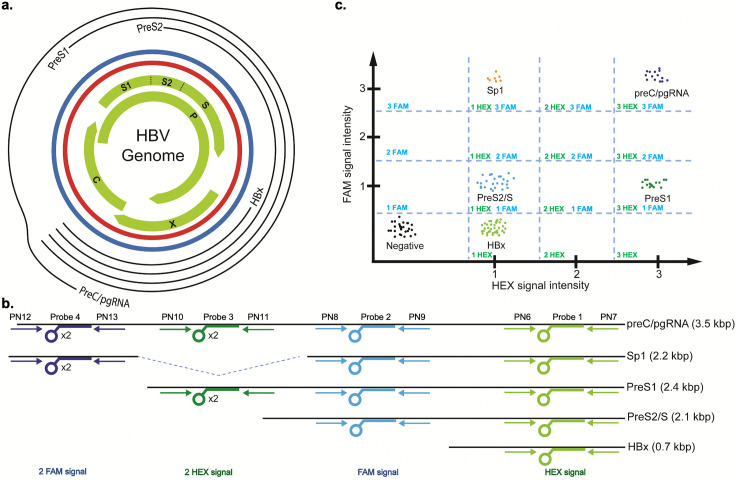
Schematic representation of ddOTs. **(A)** Schematic representation of the HBV genome with the main ORFs and the HBV RNA transcripts **(B)** Schematic representation of primers and probe locations on the 5 targeted HBV mRNA species. **(C)** Theoretical visualization on 2D-scatterplot of ddPCR results using the primers and probes represented in **B.** X-axis represent HEX signals, Y-axis represent FAM signals. Black dots represent signal-empty droplets. Each coloured dot corresponds to a droplet giving a positive signal for the indicated RNA. Probe 4 and probe 3 concentrations are doubled compared to the two other probes, giving more FAM or HEX signal intensity and inducing a shift on the 2D ddPCR plot.

Despite the existence of a prophylactic vaccine, Hepatitis B virus (HBV) infection remains a global health challenge, with an estimated 260 million individuals chronically infected and at high risk for developing severe liver diseases such as cirrhosis, liver failure or hepatocellular carcinoma. Chronic hepatitis is responsible for over 1 100 000 deaths annually [1] (WHO, report 2024). While current therapies suppress viral replication and improve liver function, they fail to eradicate the virus and thus cannot eliminate the risk of liver disease development. Therapeutic failure is partly due to the inability of existing drugs to target the viral episomal covalently closed circular DNA (cccDNA) that persists in the nuclei of infected hepatocytes. This approximately 3.2kb cccDNA serves as the template for all viral transcripts including the pregenomic RNA (pgRNA), which is encapsidated in the cytoplasm and retrotranscribed into viral DNA, a key driver of viral persistence. Understanding cccDNA biology, particularly the mechanisms regulating its transcription, is essential for designing novel therapies targeting the HBV reservoir. cccDNA transcriptional activity can be monitored by quantifying HBV RNAs in the cytoplasm of infected cells. Recent findings suggest that HBV RNA species in patient serum may serve as bio-markers for cccDNA persistence and expression during infection and treatment, underscoring the importance of precise HBV RNAs quantification for both basic research and clinical management [2–9]. cccDNA produces 5 major viral RNAs: 1) a 3.5 kb RNA that consists of two separate RNAs of almost similar size the preCore RNA (preC) and pregenomic RNA (pgRNA) referred in the text as preC/pgRNA, 2) a 2.4 kb large surface protein RNA (PreS1), 3) a 2.1 kb middle and small surface protein RNAs (PreS2 and S), 4) a 0.7 kb X protein RNA (HBx), and 5) splice variants, with Sp1 being the most expressed [10–14]. Due to its small size, the entire viral genome is coding. HBV transcripts are overlapping either completely or partially and share a common polyadenylation site and 3’ end (Fig 1), complicating individual RNA quantification. Current methods face limitations [2,15,16]. Reverse transcription quantitative PCR (RT-qPCR) can distinguish the 3.5-kb preC/pgRNA but requires sequential amplification and subtraction steps prone to primer bias [15]. Northern blotting allows semi-quantification of the main HBV transcripts but lacks sensitivity and requires large quantities of RNA. 5’RACE excludes certain transcripts and is not fully quantitative [2]. Finally, long read sequencing coupled with a capture step is promising but may miss short RNAs, requires substantial input material, and is costly. Thus, none of these technics allow a rapid, clear and sensitive quantification of the different HBV transcripts in a single reliable experiment.

To address these challenges, we developed a multiplex droplet digital PCR (ddPCR) assay named ddPCR for Overlapping Transcripts (ddOTs) for simultaneous and precise quantification of all major HBV RNA species (HBx, PreS2/S, PreS1, preC/pgRNA, and Sp1) during infection. ddPCR provides absolute quantification with higher precision and lower technical variability than qPCR [17,18], and has proven effective in pathogen diagnostics. We validated our method using synthetic HBV-like DNA templates to ensure specificity and accuracy, then applied it to quantify viral RNA dynamics in HBV-expressing cellular models including primary human hepatocytes and liver-humanized mouse models.

## Results

### Validation of ddOTs using DNA fragments covering the main HBV ORFs

**Fig 2:**
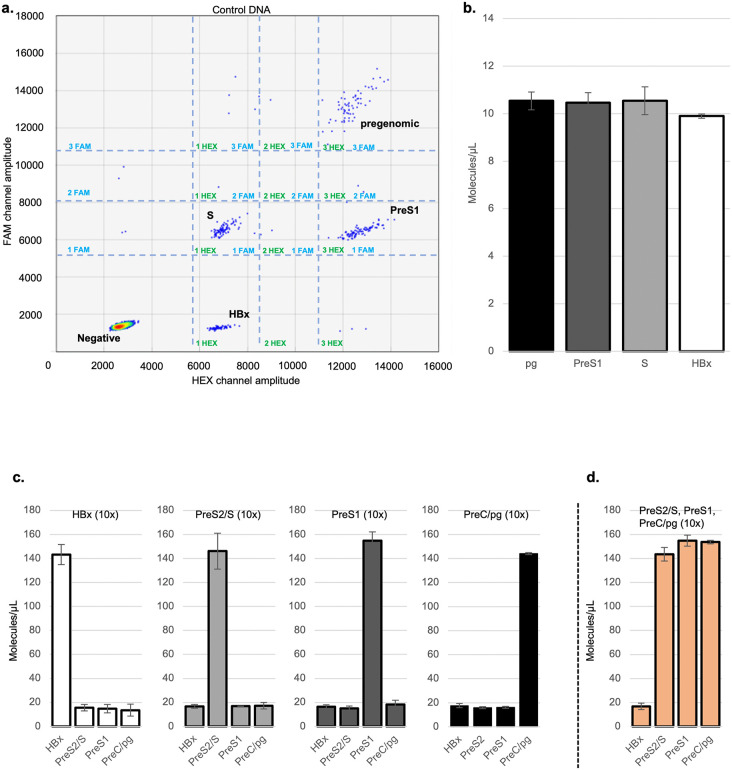
Validation of ddOTs quantification and specificity using synthetic DNAs fragments covering the 4 HBV ORFs. **(A)** to **(B)** Indicated PCR-amplified and purified HBV fragments were mixed at a concentration of 10 molecules/µl each and then quantified using ddOTs. **(A)** 2D ddPCR plot of synthetic DNA fragments HBx, PreS2, PreS1, and pregenomic quantification on QX Manager Software. X-axis represents HEX signal amplitude; Y-axis represents FAM signal amplitude. Clouds corresponding to the different HBV fragments are indicated. Sparse unannotated dots correspond to sheared molecules. Negative droplets are indicated **(B)** Absolute quantification of each HBV fragments present in samples measured by ddOTs. Errors bars represent SEM of three independent experiments. **(C)** Quantification by multiplexed ddPCR of the 4 HBV DNA fragments present at different concentration. As indicated on each graph, DNA fragments were mixed at a concentration of 15 molecules/µL each excepted for the indicated fragment added at a concentration of 150 molecules/µl (10 times more concentrated). Errors bars represent SEM of three independent experiments. **(D)** Same as in **(C)** except that all the DNA fragments are mixed at a concentration of 150 molecules/µl each except HBx DNA fragment added at a concentration of 15 molecules/µL. Errors bars represent SEM of three independent experiments.

To specifically quantify each of the overlapped HBV RNAs, we developed a multiplexed ddPCR approach. We designed four pairs of primers/probes sets targeting the 4 main HBV RNAs (HBx, PreS2/S, PreS1 and preC/pgRNA) (Fig 1B). Following reverse-transcription of RNA and partitioning of cDNA into droplets, individual cDNA molecules are simultaneously analyzed with the 4 primer/probe sets, generating a specific fluorescence profile (Fig 1C). To validate the HBV multiplexed ddPCR strategy, we first used individual purified DNA fragments spanning the 4 HBV ORFs. By testing known concentrations of each DNA fragment individually, we confirmed accurate amplification, quantification, and fluorescence profiles (S2 Fig). Evidenced by droplets outside expected clusters, shearing occurred at low levels (S2 Fig). Terminal dilution assays demonstrated linear quantification (R^2^ = 0.9989) and sensitivity down to 0.4 copies/µL (S3 Fig). We validated the assay using a mixture containing equal amounts (10 molecules/µL) of all 4 HBV DNA fragments. The 2D projection (Fig 2) shows distinct clusters for each HBV DNA, with negative droplets labeled accordingly. Absolute quantification confirmed the specificity and accuracy of the primer/probe sets, with <6% of positive droplets corresponding to sheared DNA (Fig 2; S2 Fig).

To assess performance under imbalanced conditions, we tested samples where one DNA fragment was 10-fold more concentrated than the others. The multiplexed ddPCR accurately quantified all targets regardless of overrepresentation (Fig 2C). Similarly, when HBx DNA was underrepresented, all species were reliably measured (Fig 2D). Altogether, the multiplexed ddPCR assay robustly quantifies HBV DNA fragments corresponding to major ORFs.

### Analysis of HBV RNA expression in HepAD38 cells by ddOTs

**Fig 3:**
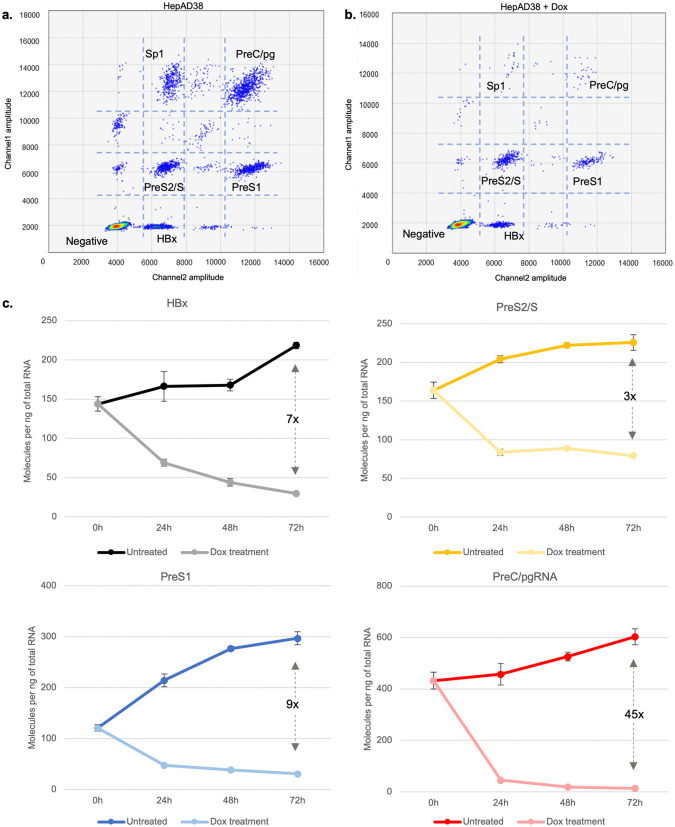
Quantification of HBV RNAs levels in HepAD38 cells treated or not with Doxycycline. HBV RNAs expression in HepAD38 cells has been quantified by ddOTs over 72-hours time-course experiment following treatment with or without Doxycycline (0.3µg/mL). 0h corresponds to the time when the cells were plated with or without Doxycycline treatment. Total RNA was extracted at different time points after plating and quantified by ddOTs. **(A)** Visualization of the 2-D ddPCR plot showing the detection of each HBV RNAs in non-treated HepAD38 cells 48h after plating. **(B)** 2-D ddPCR plot showing the detection of each HBV RNAs in HepAD38 cells treated with Doxycycline for 48h. **(C)** Quantification of the indicated HBV RNAs level at the indicated time after plating using ddOTs in HepaAD38 cells treated or not with doxycycline. Results are expressed as the number of copies of indicated RNA/ng of total RNA. Mean ± SEM of 3 experiments is shown.

Having established the conditions for the multiplexed ddPCR, we validated the assay using HBV RNA expressing cells. HepAD38 cells are derived from the HepG2 hepatoma cell line and contain an integrated cDNA copy of the HBV pgRNA under tetracycline-controlled expression. The promoter region comprises a minimal CMV promoter (-53 relative to the start site) and heptamerized upstream tet-operators. In the absence of tetracycline, this promoter drives a strong pgRNA expression, while its activity, and to a lesser extend the expression of other HBV transcripts, is silenced upon tetracycline treatment [19]. We first quantified HBV RNA molecules in untreated HepAD38 cells using multiplexed ddPCR (Fig 3A and 3C). The 2D representation in Fig 3A shows the expression levels of the 4 main HBV RNAs: HBx, PreS2/S, PreS1, and preC/pgRNA, with the latter being the most abundant, consistent with other studies [19,20]. Notably, the Sp1 splice variant was detected at relatively high levels, representing 14% of total HBV transcripts here. This aligns with reports that Sp1 constitutes up to 17% of HBV transcripts [10–14,21,22]. Additional clusters observed in the assay may correspond to other spliced HBV RNA species, which are known to vary in abundance across cell lines [23]. While RNA degradation or shearing could partially explain these extra clusters, the combined signal from the four main RNAs and Sp1 accounted for ~95% of the total, leaving only 5% attributable to minor splice variants or fragmented RNA.

To evaluate the assay-specificity for detecting oriented transcriptional changes, we analyzed HBV RNAs expression in HepAD38 cells after silencing the tet-CMV promoter with doxycycline. HBV RNA levels were quantified at multiple time points post-treatment. Fig 3B and 3C reveal a sharp decline in pgRNA and Sp1 levels within 24 hours of doxycycline exposure. HBx, PreS2/S, and PreS1 RNAs also decreased, albeit to a much lesser extent. Although these genes are regulated by their own promoters, the tet operator’s repressive effect may propagate across the HBV genome, as previously suggested [20]. However, residual expression of HBx, PreS2/S, and PreS1 RNAs might originate from cccDNA transcription, which accumulates in HepAD38 cells cultured without tetracycline. To further investigate HBV RNA re-expression, HepAD38 cells were cultured in the presence of doxycycline for four weeks prior to its withdrawal (S4 Fig). Under these conditions, low levels of HBx, PreS2/S, and PreS1 RNAs were still detectable, but their expression increased 48 hours after doxycycline removal. While pgRNA expression was markedly lower than that of other HBV RNAs in cells maintained in doxycycline, it exhibited a much stronger increase 48 hours after doxycycline withdrawal. This pronounced dynamic is consistent with pgRNA being under the direct control of the tet operator.

In conclusion, our multiplexed ddPCR assay robustly detects specific variations in HBV RNA expression, enabling precise tracking of transcriptional changes.

### Analysis of HBx RNA expression level in HepAD38 cells by ddOTs

**Fig 4:**
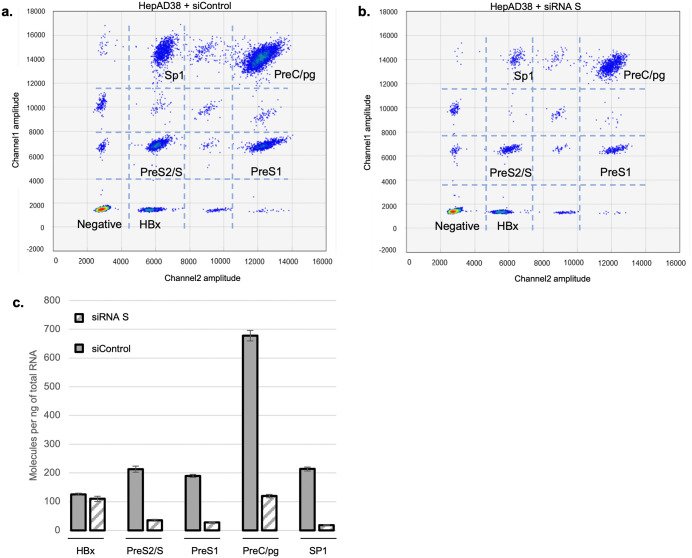
HBV RNA levels quantification in HepAD38 cells transfected with siRNA targeting the S region. HepAD38 cells were transfected with an siRNA targeting a region in S ORF that overlaps with preC/pgRNA, Sp1, PreS1 and Pres2/S HBV RNAs or with a control siRNA. 72 hours after transfection, HBV RNA levels were quantified by ddOTs. **(A)** **(B)** 2-D plot of ddPCR showing the detection of the different HBV RNAs in HepAD38 cells transfected with control siRNA (A) or with the siRNA targeting the S region **(B)**. **(C)** Quantification of the number of copies of the indicated HBV RNAs in HepAD38 cells transfected with the siRNA S or with siControl by ddOTs. Errors bars represent the SEM of three independent experiments.

Depending on the study and the technique used to assess HBV mRNAs expression, the level of HBx mRNA in HBV replicating cells varies widely, ranging from negligible to as high as PreS2 mRNA levels [2,4,15,24–26]. Our results suggested that in HepAD38 cells, HBx mRNA levels are comparable to PreS2/S or preS1 mRNA levels. Since HBV RNA quantification relies on reverse transcription of viral RNAs followed by amplification via ddPCR, one may question whether incomplete reverse transcription of longer, more abundant HBV mRNAs (e.g., PreS2 or preC/pgRNA) could generate shorter cDNAs that will be recognized and quantified as HBx mRNA. Similarly, degradation of longer HBV mRNAs may lead to erroneous quantification of HBx mRNAs. To rule this out, we measured HBx RNA levels in HepAD38 cells with or without siRNA-mediated silencing of all HBV transcript except HBx. The siRNA targeted the S ORF region. As shown in Fig 4, siRNA targeting the S region induced a ~ 80% reduction in PreS2/S, PreS1, preC/pgRNA, and Sp1 mRNA levels, while HBx RNA levels remained unchanged. As a control, transfection of HBx-targeting siRNAs directed against a region shared by all HBV RNAs led to efficient silencing of all HBV transcripts in HepAD38 cells (S5 Fig). These results validate the ddOTs approach for absolute quantification of all HBV RNAs, including HBx RNA.

### Analysis of HBV RNA expression during infection using ddOTs

The HBx protein has been shown to be required for HBV cccDNA transcription [27–29]. HBx acts primarily by degrading the SMC5/6 complex, which silences HBV transcription via a mechanism that is not yet fully understood but results in the establishment of a repressed chromatin state. A recent publication suggests that in the absence of HBx, cccDNA can still transcribe HBx mRNA [26]. To assess whether HBV promoters were differentially affected in absence of HBx, we analyzed the expression of HBV mRNAs in HepG2-NTCP cells and primary human hepatocytes (PHH) infected with wild type (HBV WT) or HBV deficient for HBx expression (HBV X-).

**Fig 5:**
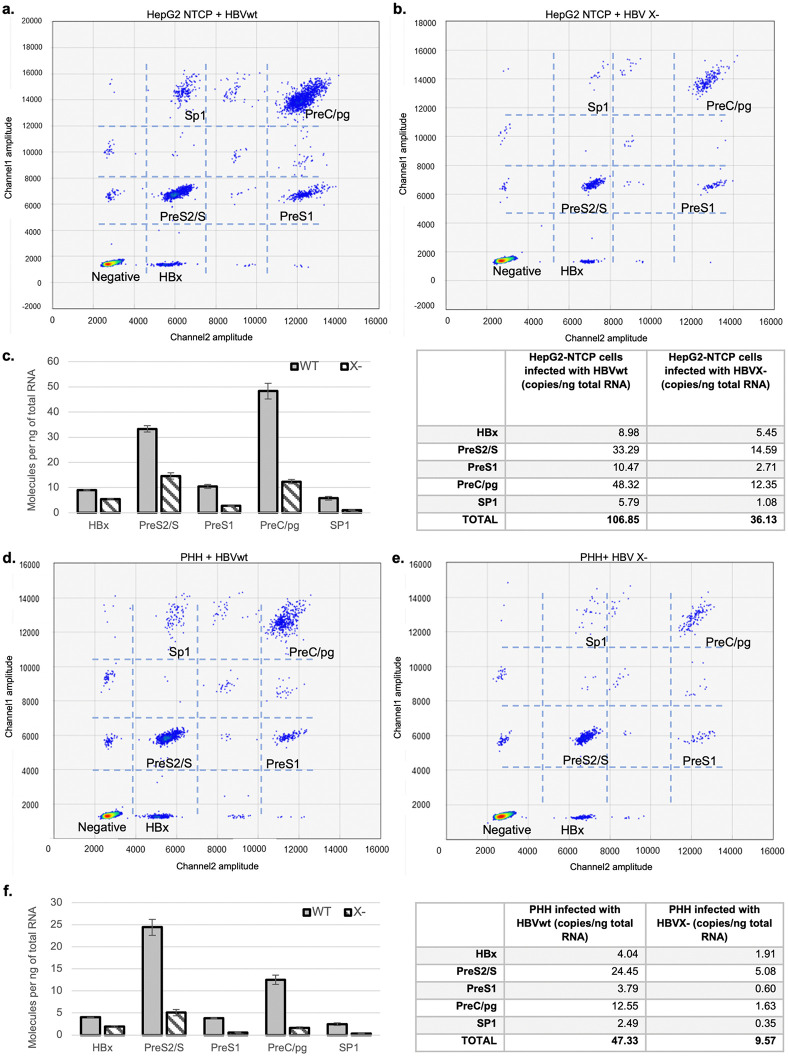
HBV RNA levels in HepG2-NTCP cells or human primary hepatocytes infected with WT or X- HBV. **(A)** to **(B)** HepG2-NTCP cells were infected with WT or X - HBV at a MOI of 100 vge/cells. Total RNA was collected 6 dpi and HBV RNAs were quantified by ddOTs. **(A) (B)** 2D ddPCR plot of HBV RNAs analysis in HepG2-NTCP cells infected with HBV WT **(A)** or HBV X- **(B)**. X-axis represents Channel 2 amplitude corresponding to HEX signals, Y-axis represents Channel 1 amplitude corresponding to FAM signals. **(C)** Absolute quantification of the different HBV RNA species in cells infected by WT or X- viruses. Results were normalized to the number of cells and expressed as number of copies per ng of total RNA. Errors bars represent the SEM of three independent experiments. **(D)** to **(F).** PHH were infected with WT or X - HBV at a MOI of 100 vge/cells. Total RNA was collected 5 dpi and HBV RNAs were quantified by ddOTs. **(D) (E)** 2D ddPCR plot of HBV RNAs analysis in PHH cells infected with HBV WT **(D)** or HBV X- **(E)**. X-axis represents Channel 2 amplitude corresponding to HEX signals, Y-axis represents Channel 1 amplitude corresponding to FAM signals. **(F)** Absolute quantification of the different HBV RNA species in cells infected by WT or X- viruses. Results were normalized to the number of cells and expressed as number of copies per ng of total RNA. Errors bars represent the SEM of three independent experiments.

As shown in Fig 5A-5C, in the presence of HBx, all HBV mRNAs were detected, with preC/pgRNA and PreS2/S being the most expressed. As observed in HepAD38 cells, the Sp1 splice variant was also detected in relatively high quantities. In HepG2-NTCP cells infected with HBV X-, the levels of all HBV RNA species decrease, while the cccDNA level remained comparable to those observed following infection with wild-type HBV, as previously reported [27,30] and shown in S6A Fig. Specifically, preC/pgRNA and PreS1 levels decrease by 3.9-fold, whereas preS2/S and HBx RNAs decrease by 2.3-fold and 1.6-fold, respectively. In infected PHH (Fig 5D-5F), all HBV mRNAs and Sp1 splice variant were again detected, with PreS2/S remaining the most highly expressed. In PHH infected with HBV X-, transcript levels decrease compared to those infected with HBV WT: preC/pgRNA and Sp1 decrease by 7-fold, PreS1 by 6-fold, PreS2/S by 5-fold, and HBx by 2-fold. In both cell lines, Sp1 expression decreased proportionally to the reduction in total preC/pgRNA plus Sp1 suggesting that transcriptional repression imposed by SMC5/6 does not impact splicing.

To assess the specificity of ddOTs, control experiments were performed in non-infected HepG2-NTCP cells. No signal was detected with ddOTs in these cells, whereas amplification of the cellular Rhot2 transcript confirmed RNA integrity and successful reverse transcription (S6B Fig). Furthermore, no signal was observed when ddOTs were performed in the absence of reverse transcription (-RT) in either infected HepG2-NTCP cells or PHH, confirming the absence of contaminating DNA (S6C Fig).

Overall, our results validate the use of ddOTs to quantify HBV transcription in the context of infection and suggests that HBV promoters are differentially affected in the absence of the HBx protein.

### Analysis of dynamics of HBV RNA expression in early stages of infection by ddOTs

**Fig 6:**
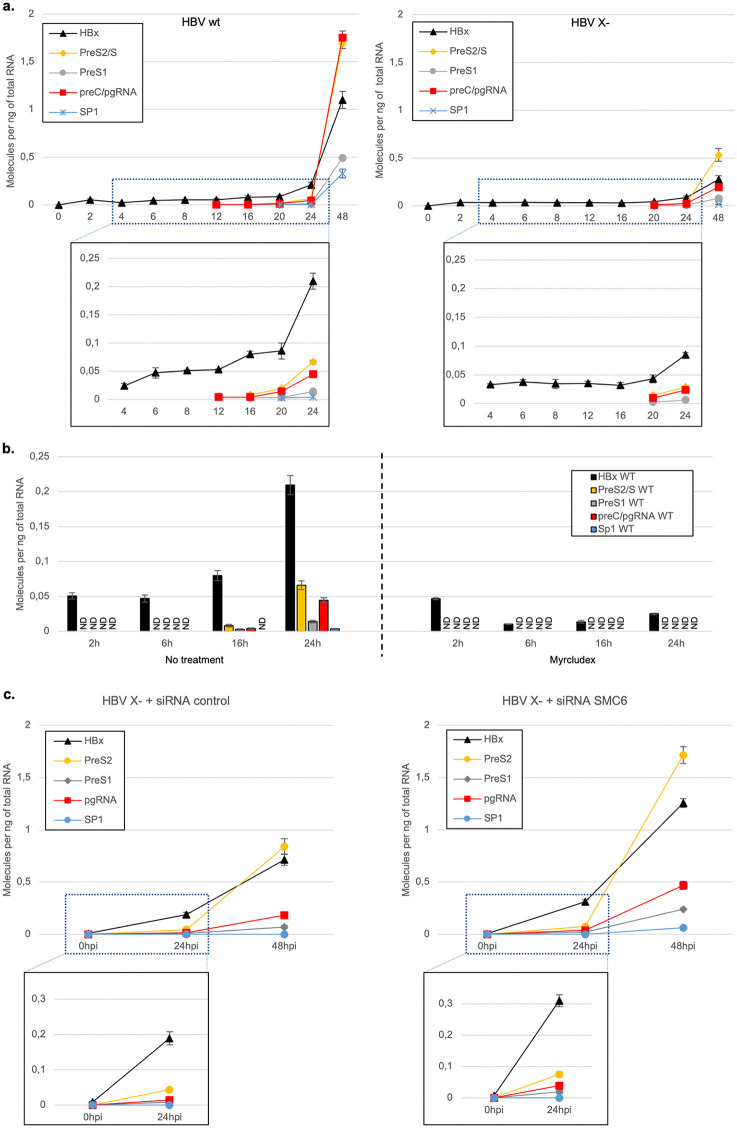
Analysis of HBV RNAs kinetic in HepG2-NTCP cells using ddOTs. **(A)** and **(B)** HepG2-NTCP cells were infected with spinoculation of WT or X - HBV at a MOI of 100 MOI vge/cells and total RNA was extracted at the indicated time after infection. **(A)** Absolute quantification by ddOTs of the 4 main HBV RNAs and Sp1 in cells infected with WT (left) or X- (right) viruses. **(B)** Absolute quantification by ddOTs of the 4 main HBV RNAs and Sp1 in HepG2-NTCP cells treated or not for 2 hours with 500nM Myrcludex and infected with HBV WT at a MOI of 100 MOI vge/cells. **(C)** Absolute quantification by ddOTs of the 4 main HBV RNAs and Sp1 in HepG2-NTCP cells transfected with an siRNA control (left panel) or targeting SMC6 (right panel) and infected with HBV X- at a MOI of 100 MOI vge/cells. Total RNA was extracted at the indicated time after infection. Results are expressed as number of copies per ng of total RNA. Errors bars represent the SEM of three independent experiments.

The dynamics of HBV RNA expression during early steps of infection remains unclear. Studies suggest that HBx RNA is present in the incoming viral particle and is translated, leading to the degradation of SMC5/SMC6 and consequently initiating transcription from cccDNA, while other studies propose that HBx RNA is expressed before other HBV RNAs in order to enable cccDNA transcription [31,32]. Finally, Lucifora and colleagues proposed that repression is established shortly after cccDNA transcription begins, allowing the expression of HBV RNAs including HBx RNA [27]. To investigate the kinetics of HBV RNA expression and determine whether HBx RNA is an early transcript, we infected HepG2-NTCP cells with HBV WT or HBV X- viruses and quantified HBV RNA levels at different time-points post-infection as well as cccDNA level at 48h after infection (Figs 6A and S7A). HBx RNA is detected as early as 2 hours post-infection (h.p.i) with a slight increase from 4h to 12 h.p.i, coinciding with the initial detection of preC/pgRNAs. A pronounced increase in HBx RNA occurred at 16 h.p.i., simultaneous with the detection of all HBV RNA species (Figs 6A, 12–16 h.p.i.). Between 16–24 h.p.i., all HBV RNAs showed a steady increase, though preC/pgRNA, preS2, and preS1 RNAs exhibited far greater amplification (33-fold in average), compared to HBx RNA increase (5-fold) from 24 to 48 h.p.i. In HBV X- infected cells, HBx RNAs were detected early but remained constant until 16 h.p.i. All HBV RNA species emerged concomitantly at 20 h.p.i., coinciding with a rise in HBx RNA. Unlike HBV WT, HBV X− transcription remained low over time, confirming HBx’s necessity for sustained cccDNA transcription. Notably, while HBV RNA levels were similar between WT and X− viruses at transcriptional onset, HBV X− transcription was delayed (20 h.p.i. vs. 16 h.p.i.) and failed to amplify over time. Furthermore, HBV X− infection skewed RNA abundance: preS2/S RNA dominated, unlike WT infections where preC/pgRNA and preS2/S RNA were predominant, suggesting that HBV promoters are regulated differentially in response of the cellular environment.

To test if HBx transcripts detected during the first hours after infection originate from inoculum-derived enveloped particles, we pretreated HepG2-NTCP cells with myrcludex, which block the NTCP receptor, 2 hours before infection. In treated cells, HBx RNAs decreased rapidly by 2 h.p.i., with no subsequent viral transcripts detected. In contrast, untreated cells showed rising HBx RNA levels by 2 h.p.i., indicative of neo-transcribed RNA (Fig 6B). This suggests a portion of early HBx RNA enters cells via NTCP-independent routes [2,33], while subsequent transcription requires NTCP-mediated entry.

To further assess whether spinoculation contributes to the early detection of HBx RNA, we infected HepG2-NTCP cells with HBV WT in the absence of spinoculation and quantified HBV RNA levels at different time points post-infection (S7B Fig). Residual HBx RNA was again detected, and the kinetics of appearance of the different HBV RNA species remained comparable to those observed in Fig 6. These results indicate that spinoculation does not influence the early detection of HBx RNA.

Finally, we investigated whether silencing SMC5/6, thereby mimicking HBx function, could restore both the kinetics and expression levels of HBV RNAs. To this end, HepG2-NTCP cells transfected with siRNA targeting SMC6 or control siRNA were subsequently infected with HBV X^−^, and HBV RNA levels were quantified at different time points post-infection (Figs 6C and S7C). Silencing of SMC6 restored both the expression levels and the kinetics of HBV RNA production.

Altogether, these results validate ddOTs for sensitive quantification of HBV transcripts, even at low-abundance stages. They confirm HBx RNA’s early detection, its essential role in sustaining cccDNA transcription, and differential regulation of HBV promoters. While incoming HBx RNA is detectable, its functional significance in HBV replication warrants further study.

### Studying HBV RNA expression in HBV-infected human liver chimeric mice

**Fig 7:**
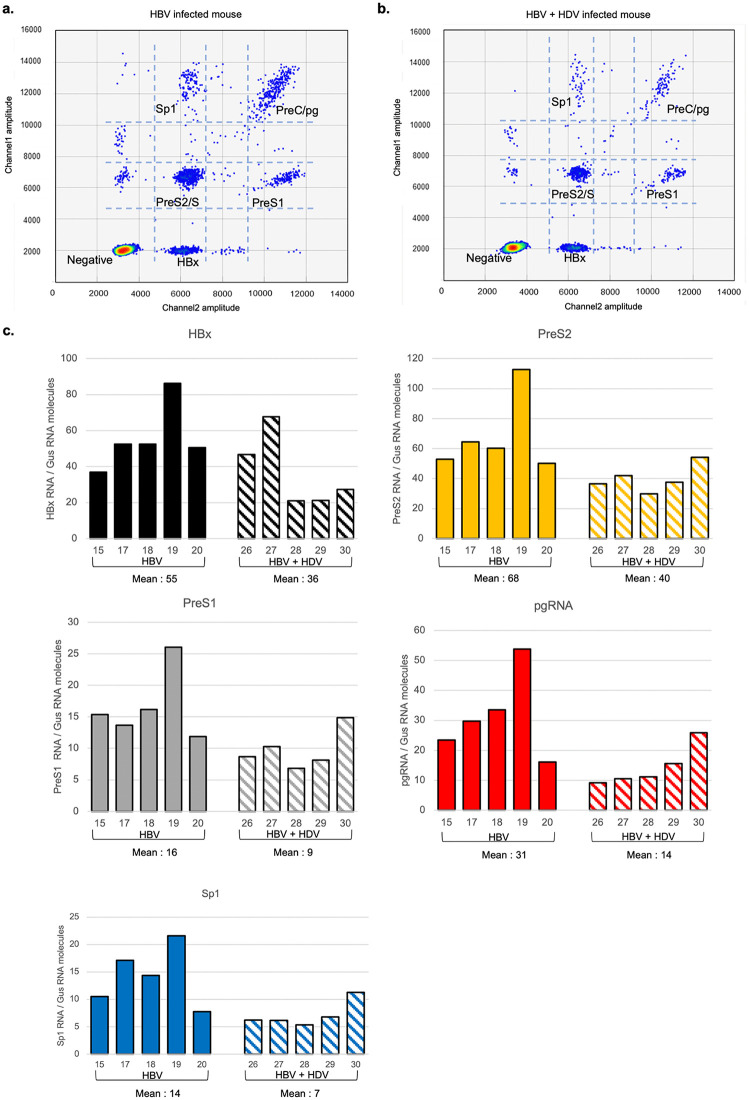
HBV RNA levels quantification in liver-humanized mice infected with HBV alone or coinfected with HBV and HDV. FRG liver-humanized mice (HuHep) were infected either with HBV alone or with both HBV and HDV. Eight weeks after infection (wpi) sera and the liver tissues were collected for viremia analysis and intrahepatic total RNA extraction respectively. **(A)** Representative 2-D ddPCR plot showing the detection of intrahepatic HBV RNAs in the liver of an HBV mono-infected humanized mouse. **(B)** Representative 2-D ddPCR plot showing the detection of intrahepatic HBV RNAs in the liver of an HBV/HDV co-infected humanized mouse. **(C)** Absolute quantification of the different HBV RNA species in HBV mono-infected and HBV/HDV co-infected HuHep mice. Each mouse is identified by a number: 15, 17, 18, 19, and 20 for HBV mono-infected mice; and 26, 27, 28, 29, and 30 for HBV/HDV co-infected mice. Results are expressed as the number of RNA copies normalized to human GUS (hGUS) RNA expression.

To further validate our assay using *in vivo*-infected hepatocytes, we used the chimeric human liver FRG mouse model (HuHep mice). HuHep mice were either infected with HBV alone or co-infected with HBV and HDV. Eight weeks post-infection, viremia was assessed, and livers from HBV-infected mice (5 mice) or HBV and HDV co-infected mice (5 mice) were harvested for RNA isolation (Figs 7 and S8A). As shown in Fig 7, all HBV mRNAs were detected, with PreS2/S and preC/pgRNA being the most abundant, in agreement with previously published results obtained in HBV-infected human liver chimeric mice [34]. Moreover, we observed a decrease in HBV RNA levels in HBV/HDV co-infected livers, consistent with published data [35]. We next compared the expression profiles of preC/pgRNA + Sp1, as well as total HBV RNAs, in mono-infected and HBV and HDV co-infected HuHep mice using ddOTs and RT-qPCR (S8 Fig). Both approaches yielded highly comparable expression patterns, further supporting the robustness of our method.

Overall, our data validate ddOTs as a reliable approach for quantifying HBV RNAs *in vivo* and support its use in frozen tissue samples.

## Discussion

The compact structure of the HBV genome and the overlap of different open reading frames have been a long-time roadblock for the development of a quantitative affordable daily usage assay for understanding HBV transcription dynamics. In this study, we developed the ddOTs method to quantify the major HBV RNAs: preC/pgRNA, PreS1, PreS2/S, HBx and Sp1. Using DNA covering the four HBV ORFs we first showed that we were able to quantify accurately each of the DNA species even under imbalanced conditions. We next validated this approach using different models of HBV RNA expression. Using ddOTs we were able to achieve absolute quantification of the 4 main HBV RNAs (preC/pg, PreS1, PreS2/S, HBx) as well as the splice variant Sp1. Using siRNAs targeting the S region and thus decreasing the expression of preC/pgRNA, PreS1, PreS2/S and Sp1 RNAs, we showed that ddOTs allows the specific quantification of the HBV X RNAs. Finally using different settings known to modulate differentially HBV transcription (i.e., treatment of HepAD38 cells with doxycycline or infection of HepG2-NTCP cells or PHH with HBV X- virus or HuHep mice infected by HBV alone or co-infected with HBV and HDV) or performing time course analysis, we demonstrated that ddOTs can be used to accurately quantify oriented transcriptional changes.

Using terminal dilution assays, we showed that ddOTs can detect as few as 0.4 copies/µL using DNA fragments covering the HBV ORFs. It will thus be possible to use ddOTs to analyze HBV transcription in limited materials such as patient’s biopsies. However, our technic’s efficiency is directly dependent of the quality of the RNA and may require standardized tissue-banking methods allowing high quality RNA extraction. Moreover, our method has been developed for the detection of genotype D HBV RNAs and should thus be further adapted for the detection of the different HBV genotypes (S9 Fig). Indeed, mismatch effects in PCR amplification depend on their position and type. Mismatches at the 3′ end of primers are more detrimental than those at the 5′ end. Among mismatch types, purine-purine mismatches have the strongest negative impact. However, fewer than three mismatches within a primer generally do not significantly affect amplification in end-point reactions such as ddPCR. For probes, mismatches are more harmful when located in the center of the sequence [36–39]. Sequencing of the HBV genome cloned in HepAD38 cells revealed similarities to genotype D3 with only minor mismatches relative to the primers/probe sets used for ddOTs (S9 Fig). Consistent with previous studies, HBV RNAs in HepAD38 cells were efficiently amplified. However, detecting other HBV genotypes with more mismatches in primers or probes will require either genotype-specific primers or the use of degenerate primers and probes, similar to strategies developed for HIV reservoir quantification [40]. The usability of degenerate primers for multiplex ddPCR amplifications has not been investigated yet and may be an easy solution for detection of HBV RNA of any genotype.

HBx RNA expression level reported in the literature is highly variable depending on the technics used to detect/quantify HBV RNAs. To further demonstrate that ddOTs specifically quantify HBx RNA and not degradation products of larger HBV RNAs, we induced the degradation of all the HBV RNA except HBx using an siRNA targeting the S ORF and we showed that the level of HBx remain constant. Our results are in line with previous studies showing that HBx mRNA is expressed at a level comparable to those of structural genes and remains relatively constant over time [2,26,32,41]. Discrepancy with other studies may come from technical caveat as the removal of short transcripts during sample preparation for long read sequencing or the variation in PCR amplification of the different HBV regions when using a subtractive based RT-PCR assay [15,16].

Using ddOTs we were able to clearly identify and quantify simultaneously the 4 main HBV transcripts and Sp1 splice variant in HepAD38, HepG2-NTCP cells, PHH and in liver samples from infected HuHep mice. The relative abundance of each RNA species is in line with previous observations, i.e. preC/pg RNA being reported as the most abundant HBV transcripts in HepG2-NTCP cells and PreS2/S in PHH while PreS2/S and preC/pgRNA seems to be the more abundant transcripts in chimeric mice [26,34]. Sp1 is the most abundant splice variant in every cell line we tested, which is also consistent with published results [10–13]. Beside Sp1, we were also able to identify additional clouds that could represent the different spliced RNAs described in the literature [23,42,43]. Recent studies suggest that spliced HBV RNA can represent up to 30% of total RNAs. Lim *et al.* [23] showed a high spectrum of splice variants present in HBV-infected cells and patients by analyzing more than 500 RNA-seq libraries. However, we cannot completely rule out that these additional clouds may in part be due to HBV RNA degradation or shearing. The development of probes with new fluorochromes will improve the identification of additional spliced variants. To this day, limitations of Bio-Rad QX Manager software do not permit to quantify molecules simultaneously bound by more than 3 different fluorochromes. Once this software limitation is addressed, the design of new primers/probe sets with different fluorescence will allow better separation and discrimination between transcripts, such as PreS2 and S or between splice variants. ddOTs methodology may thus be applied to other compact-genome systems that present overlapping transcript sequences, such as prokaryotes, viruses, and eukaryotes [44–46].

The relevance of ddOTs to study the transcriptional regulation of the HBV promoters in the context of the full HBV genome is first supported by our results in HepAD38 cells that contain a cDNA copy of the HBV pgRNA. Using ddOTs we were able to quantify simultaneously and independently the expression of the 4 main HBV RNAs and of Sp1 in cells treated or not with doxycycline over a period of 72h. We clearly observed a differential sensitivity to doxycycline treatment of the different promoters with a strong decrease of more than 45-fold for the HBV pgRNA directly controlled by the tet CMV promoter compared to preS1, preS2/S and HBx RNAs that are regulated by their own promoter. In a more physiologically relevant system, we were able to assess the regulation of the 4 main HBV promoters individually using HepG2-NTCP cells or PHH infected by HBV WT or HBV X- viruses. In accordance with the literature, the expression of HBV RNAs was strongly repressed in the absence of HBx expression. However, our results suggest that the HBV promoters do not respond similarly to SMC5/SMC6 silencing. Recently Peng and collaborators, using 5’-terminal single-cell sequencing analysis, showed that while HBV RNAs expression is strongly decreased in human primary hepatocytes infected by HBV X-, some HBV RNAs are still expressed, albeit at low level, and the majority of them correspond to long HBx RNA starting at a non-conventional TSS in the enhancer 1 [26]. These results suggest that HBV promoters are differentially regulated. In line with these findings, recent results from Prescott and collaborators show that HBx RNA expression strictly relies on chromatin assembly at HBx promoter compared to the other HBV promoters. While regulatory elements present in the HBV promoters and enhancers have been well characterized, little is known yet on their regulation in the context of the cccDNA in the cell. ddOTs is thus a promising tool to study HBV transcriptional and post-transcriptional regulation.

Using ddOTs we assessed the kinetics of HBV RNAs expression and showed that HBx RNAs are detectable very early after infection. While part of this HBx RNAs, as suggested by others, are delivered from the inoculum, they however seem to enter the cell through an NTCP-independent mechanism. Moreover, their level decreased quite rapidly over time (6h post infection), as shown in myrcludex treated cells. One may ask the significance and the role of these incoming HBx RNA in virus replication. It has been suggested that they serve as the template for the translation of HBx proteins allowing cccDNA transcription through degradation of SMC5/SMC6. Others suggest however that these incoming HBx RNA are not really required for the priming of cccDNA transcription and favor a model where HBx RNAs are the first RNA to be transcribed from established cccDNA, allowing the subsequent transcription of all the HBV viral genes [32,41]. In our study, we observed that contrary to myrcludex treated cells, in untreated cells HBx RNAs increase suggesting that they are neo-transcribed from the freshly established cccDNA. To confirm this, it will be interesting to block transcription before infection to assess the contribution of cccDNA transcription to those early HBx RNAs.

In conclusion, this novel ddOTs technique is an easy to implement and rapid assay allowing simultaneous quantification of the main HBV RNAs individually, in an accurate, reliable and highly reproducible fashion. The cost-effectiveness and speed of the procedure make it a very easy and universal way to assess the dynamics and regulation of HBV promoters, and the regulation of HBV RNA expression with unprecedented specificity. This approach may also prove useful to quantify and identify HBV circulating RNAs that could represent a powerful indicator of HBV cccDNA transcription. Finally, ddOTs approach could be adapted for the study of other organism whose genomes contain overlapping genes.

## Materials and methods

### Cell culture, HBV stock production and infection

HepG2-NTCP cells were grown in DMEM-Glutamax supplemented with 10% FBS and 1% Penicillin/Streptomycin (10,000 units penicillin and 10 mg streptomycin/mL) [47]. Primary human hepatocytes were maintained in PHH medium (Corning, reference 355056, hepatocyte culture media kit, 500 ml) according to the manufacturer’s recommendations. HepAD38 cells are derived from HepG2 cells and contain an integrated HBV genome (subtype ayw) under tetracycline control [19]. HepAD38 cells were grown in DMEM F12 + Glutamax supplemented with 10% FBS, 1% Penicillin Streptomycin, Hydroxycortisone (0.2µg/mL) and Insulin (5µg/mL). When notified, 0.3µg/mL of Doxycycline was added. HepG2 H1.3∆x cells are also derived from HepG2 cells and contain an integrated 1.3 HBV genome that carries a stop codon mutation in both HBx open reading frames [27]. HepG2 H1.3∆X cells were maintained in DMEM/F-12 (Gibco) supplemented with 10% FCS, 1% (v/v) penicillin/streptomycin, 3.5 × 10^–7^ M hydrocortisone hemisuccinate (Sigma), and 5 μg/ml insulin (Sigma).

For HBV production, HepAD38 or HepG2 H1.3∆x cells were grown in Williams E medium supplemented with 5% FCS, 3.5 × 10^–7^ M hydrocortisone hemisuccinate or 7.10^-5^M hydrocortisone hemisuccinate, 5µg/mL insulin, and 2% dimethylsulfoxyde. WT and X- HBV genotype D particles were concentrated from the clarified supernatant of HepAD38 and HepG2 H1.3∆x cells respectively. Supernatants were collected after 5 days in Williams E production medium supplemented with 2% DMSO and digested with Roche DNase I for 1h at 37°C. Nucleocapsids were then concentrated by ultracentrifugation at 32 000xg for 4h on 20% sucrose cushion [27]. Titers of enveloped DNA-containing viral particles were determined by immunoprecipitation with an anti-preS1 antibody (gift from C. Sureau) followed by DNA extraction and qPCR. For infection, only enveloped DNA-containing viral particles (vp) were considered to determine the multiplicity of infection (MOI). PHH and HepG2-NTCP cells were infected by spinoculation (1000xg for 30min) at a multiplicity of infection of 100 vge/cell in respective culture media supplemented with 4% PEG (polyethylene glycol 8000) and 3% DMSO. Medium containing virus was removed the next day, cells were washed with PBS and cultured in medium supplemented with 2% DMSO until processing.

For kinetic analysis, HepG2-NTCP cells were infected by HBV either by spinoculation for 30min followed by 1h30min incubation at 37°C or by 2h incubation without spinoculation. Cells were then washed with PBS multiple times, and corresponding media supplemented with 2% DMSO was added until RNA extraction. For time-point 0, cells were covered with medium containing virus at 100 vge/cell for 1min and then washed multiple times with PBS. When required, Myrcludex was used at 500nM.

For RNA interference, HepAD38 or HepG2-NTCP cells were transfected with gene-specific siRNA at a concentration of 10nM using Lipofectamine RNAiMAX Transfection Reagent according to manufacturer’s recommendations. The siRNA sequences used are the following: PreS targeting siRNA sequence is (5’- CUUCCUAUUAACAGGCCUATT), SMC6 (5’-GCUAUUGAAUCUUGCUUAATT), HBx1 (5’-GAGGACUCUUGGACUCUCATT), HBx2 (5’-ACCUCUGCCUAAUCAUCUCUUTT) and control non-targeting siRNA sequence is (5’-UGGUUUACAUGUCGACUAATT).

### Experiments in HuHep mice

For infection of liver-humanized mice, HBV inocula were prepared from HepAD38 supernatants as described above, whereas hepatitis D virus (HDV) inocula were prepared from supernatants from co-transfected HuH7 cells as previously described [35,48]. Supernatants containing HBV or HDV particles were concentrated with 8% PEG 8000 (Sigma-Aldrich). All virus preparations were tested for the absence of endotoxin (Lonza).

All experiments in mice were performed by Yecuris Inc, a USA-based corporation that is authorized to handle liver-humanized FRG mice in level 3 animal facility and use local ethical rules for handling these animals. Mice were matched and grouped according human albumin levels. Five huFRG mice were intraperitoneally infected with HBV (1x10^8^ vge/mouse) and 5 hu-FRGmice were IP co-infected by HBV and HDV (with respectively HBV 1x10^8^ vge/mouse and HDV 1x10^7^ vge/mouse). Mice were euthanized and blood samples, as well as pieces of liver (flash frozen in nitrogen) were collected 8 weeks post-infection, stored at -80°C and processed for virologic analysis. HBV and HDV viremia were determined by extraction of DNA and RNA from sera using the NucleoSpin RNA Virus kit (Macherey Nagel) followed by qPCR or qRT-PCR with specific primers as described previously [35].

### Primers and probe design

Primers and probes design were based on HBV genotype D ayw subtype.

Primers used for PCR amplifications and ddPCR were designed using the online Primer3plus software (https://primer3plus.com/Primers). Primers were designed with special attention to the following details: (i) amplicon size between 60 and 200 bp, (ii) primers GC content between 50 and 60%, (iii) avoid repeats of Gs or Cs longer than 3 bases, (iv) when possible 3’ ends with a G or a C.

Probes for ddPCR were designed using IDT PrimerQuest online web interface (https://www.idtdna.com/Primerquest/). The probes design adhered to the following details: (i) probe must not overlap with the prime sequences, (ii) Tm should be 3–10°C higher than that of the primers, (iii) the probe should anneal to the strand that contains more Gs than Cs, (iv) the length of the probe should be less than 30 nucleotides, (v) absence of a G at the 5’ end. Probes were labeled with either 5’ FAM or 5’HEX fluorophores.

Primers PN6, PN7 and Probe 1 (referred as primers/probe set 1, Table 1) were used for the amplification and detection of a region within the HBx ORF. Primers PN8, PN9 and Probe 2 (primers/probe set 2) were used for the amplification and detection of a region within the PreS2/S ORF. Primers PN10, PN11 and Probe 3 (primers/probe set 3) were used for the amplification and quantification a region within the PreS1 ORF. Primers PN12, PN13 and Probe 4 (primers/probe set 4) were used for the amplification and quantification a region within the *preC*/pgRNA ORF (Fig 1).

**Table 1:** Primers and probes (positions are indicated relative to EcoRI site).

*Name*	*Sequence 5’ - 3’*	*Position to EcoRI*	*Usage*
PN1	GTGCACTTCGCTTCACCTCT	1581-1600	PCR, forward primer for synthesis of HBx DNA fragment
PN2	GTTGCCCGTTTGTCCTCTAATTC	465 - 487	PCR, forward primer for synthesis of PreS2 DNA fragment
PN3	GCAGAATCTTTCCACCAGCAATCCTC	2855-2880	PCR, forward primer for synthesis of PreS1 DNA fragment
PN4	TGTCAACACTAATATGGGCCTAA	2166-2188	PCR, forward primer for synthesis of pregenomic DNA fragment
PN5	GGTGAAAAAGTTGCATGGTGCTGGTGC	1805-1831	PCR, reverse primer for synthesis of HBx, PreS2, PreS1, and pg fragments
PN6	ACTTCGCTTCACCTCTGCAC	1585-1604	ddPCR, forward primer for HBx quantification
PN7	GTCGGTCGTTGACATTGCTG	1677-1696	ddPCR, reverse primer for HBx quantification
Probe 1	CACCGTGAACGCCCACCAAATATTG	1618-1642	HEX Probe complementary to HBx ORF
PN8	GCACCTGTATTCCCATCCCATC	596 - 617	ddPCR, forward primer for PreS2/S quantification
PN9	AAAGCCCTACGAACCACTGAAC	694 - 715	ddPCR, reverse primer for PreS2/S quantification
Probe 2	ATTCCTATGGGAGTGGGCCTCAGC	636 - 659	FAM Probe complementary to PreS2/S ORF
PN10	AAGGTAGGAGCTGGAGCATT	2985-3004	ddPCR, forward primer for PreS1 quantification
PN11	CTTCCTGACTGGCGATTGG	3106-3124	ddPCR, reverse primer for PreS1 quantification
Probe 3	TGCCAGCAAATCCGCCTCCTGCCTC	3079-3103	HEX Probe complementary to PreS1 ORF
PN12	AGTTCAGGCAACTCTTGTGG	2189-2208	ddPCR, forward primer for pregenomic quantification
PN13	GGGCATTTGGTGGTCTATAAGC	2293-2314	ddPCR, reverse primer for pregenomic quantification
Probe 4	TCGGAGTGTGGATTCGCACTCCTC	2267-2290	FAM Probe complementary to preC/pgRNA ORF
PN14	CCCAACTCCTCCCAGTCTTTA	1726-1746	Primer for reverse transcription
PN15	CCACCTTGGCTACCAGTTT	–	ddPCR, forward primer for Rhot2 quantification
PN16	TGCTGGGAACACACTGAAA	–	ddPCR, reverse primer for Rhot2 quantification
Probe 5	TCAACCACCTTGGCTACCAGTTTGT	–	Probe complementary to Rhot2 ORF
PN17	CCCACTCAGTAGCCAAGTCA	–	ddPCR, TaqMan. Forward primer for GusB quantification
PN18	CACAAAACCCAGGCCAGAAA	–	ddPCR, TaqMan. Reverse primer for GusB quantification
Probe 6	CGTGTCCCTTCCTCCCCGAG	–	ddPCR, TaqMan. HEX probe complementary to GusB ORF
PN19	CCGAATGTTGCCCAAGGTCT	1633 - 1652	TaqMan, forward primer for all HBV RNAs quantification
PN20	CCCCAACTCCTCCCAGTCT	1729 - 1747	TaqMan, reverse primer for all HBV RNAs quantification
Probe	TCAACGACCGACCTTGAGGCA	1685 - 1705	TaqMan, FAM Probe complementary to all HBV RNAs
PN21	GGAGTGTGGATTCGCACTCCT	2269 - 2289	TaqMan, forward primer for HBV pgRNA quantification
PN22	AGATTGAGATCTTCTGCGAC	2417 - 2436	TaqMan, reverse primer for HBV pgRNA quantification
Probe	AGGCAGGTCCCCTAGAAGAAGAACTCC	2358 - 2384	TaqMan, FAM Probe complementary to HBV pgRNA

### HBV DNA fragments

DNA fragments corresponding to the 4 HBV ORFs preC/pgRNA, PreS1, PreS2/S and HBx were synthetized by PCR using the pFC80 plasmid as template. This plasmid carries HBV genomes cloned head-to-tail into the EcoRI site of pBR322 [49]. HBx DNA fragment was generated using primers PN1 and PN5. PreS2DNA fragment was generated using PN2 and PN5. PreS1DNA fragment was generated using PN3 and PN5. pgDNA fragment covering preC/pgRNA ORF was generated using primers PN4 and PN5. PCR products were purified by gel extraction using Monarch Gel Extraction Kit following manufacturer’s protocol. Vortex steps were replaced by gentle pipetting to avoid DNA shearing. DNAs were quantified by Nanodrop (ThermoFisher Nanodrop One).

### RNA extraction

Total RNA was prepared using RNeasy Plus Mini Kit (QIAGEN). Manufacturer’s protocol was modified to preserve RNA integrity at maximum. All vortexing steps were replaced by gentle pipetting, and all centrifugations were done at 4°C and 7,000xg with soft acceleration/breaking. Samples were kept on ice throughout the process.

Briefly, 4 x 10^5^ cells were resuspended in 400µL of cold RLT Plus Buffer (supplemented with 40mM DTT) and then mixed with 150µL of QIAGEN RNA Protect Tissue Reagent (QIAGEN ID.76104). The mixture was then transferred to the gDNA eliminator column and centrifuged. 400µL of cold 70% EtOH were added to the flow-through and mixed gently by pipetting. Approximatively 750µL of the mix were transferred to RNeasy Spin Column and centrifuged. Subsequent steps were done following supplier’s protocol. To remove any DNA contamination, samples were treated with 2 units of Invitrogen Turbo DNase for 30 minutes at 37°C followed by addition of DNase inactivation reagent.

For mouse samples, livers were ground using a mortar and pestle, and 20–30 mg tissue pieces were homogenized with a Dounce homogenizer in an appropriate volume of RA1 buffer supplemented with 20 mM DTT. RNA was then purified using the NucleoSpin RNA Mini kit (MACHEREY-NAGEL), including DNase treatment according to the manufacturer’s protocol.

### RNA quality control

To ensure the accuracy of downstream ddPCR quantifications, quantity, quality, and integrity analysis of extracted RNAs were performed. Samples were processed using Agilent RNA 6000 Nano Kit to measure the RNA Integrity Number (RIN) using Agilent 2100 BioAnalyzer (Agilent Technologies) according to manufacturer’s protocol. Only samples presenting a RIN of 10 were conserved for our downstream applications; however, samples with a RIN greater than or equal to 8 are considered acceptable for ddOTs.

### Reverse transcription

Total RNA (500ng) was retrotranscribed using ThermoFischer SuperScript IV enzyme and HBV primer PN14 that binds in the 3’ region of HBx. For Rhot2 RNA quantification, total RNA was retrotranscribed using oligo(d)T. Resulting cDNA was then diluted and processed for ddPCR. For human GusB RNA quantification, total RNA was retrotranscribed using LunaScript RT SuperMix Kit (containing random hexamer and oligo-dT primers).

### TaqMan assay

HBV total RNAs and pregenomic RNA (pgRNA) were quantified using the TaqMan Fast Advanced Master Mix (Life Technologies) and normalized to GusB cDNA levels.

### ddOTs

In order to detect the different HBV RNA species using multiplexed ddPCR, we designed four pairs of primers and 4 probes that cover the four ORFs in the viral genome and allow for the discrimination and quantification within droplets via multiplexed PCR of the different cDNA species generated from the different HBV RNA species (Fig 1A and 1B). Briefly, HBx cDNA hybridizes with primers/probe set 1, giving only one HEX signal in quadrant 1 HEX/0 FAM, while cDNA from PreS2/S transcript hybridizes with 2 primers/probe sets (set 1 and 2) labelled respectively by HEX and FAM appearing in quadrant 1 HEX/1 FAM, cDNA from PreS1 is amplified simultaneously by 3 primers/probe sets (1,2 and 3) in droplets appearing then in quadrant 3 HEX/1 FAM and preC/pgRNA cDNA is amplified simultaneously by 4 primer/probe sets in droplets appearing in quadrant 3 HEX/3 FAM. cDNA synthetized from Sp1 HBV RNA is amplified by primers/probe sets 1,2 and 4 (quadrant 1 HEX/3 FAM).

Droplets with no signal are represented as “negative” on the 2D projection (Fig 1). Every other signal is considered as positive. ddPCR is a method for absolute quantification of nucleic acid concentrations through the combination of limiting dilution, end-point PCR and Poisson statistics. It is therefore important to do measurements in the right range of positive to negative ratio. Indeed, for high concentrations of positive droplets (>20% of total droplets), probability of having more than 1 molecule per droplet increases exponentially. This phenomenon can bias the measurement by overestimating the number of longer molecules, especially pgRNA molecules in our case (as example, a droplet containing Sp1 and PreS1 molecules will be detected as pgRNA). For low concentrations of positive droplets, most of the partitions will contain zero copies of the target molecules and nearly all positive partitions will contain only one copy of the target molecule. To ensure that almost all droplets contain only one molecule and the measurement remains accurate in multiplex conditions, we recommend keeping the positive to negative ratio lesser than 0.2. Samples must be diluted prior to droplet generation in order to obtain less than 20% of positive droplets.

As RNA integrity is primordial for discrimination and quantification of the different HBV RNA species by RT-ddPCR, we recommend assessing RNA Integrity using Agilent 2100 BioAnalyzer and use RNA presenting RIN over 9. In this study, all the *in vitro* experiments were performed using RNA samples with a RIN of 10.

To show the effects of low RNA quality on quantification, we voluntarily induced degradation of RNA sample by heating/vortexing and quantified RNAs by ddPCR (S1 Fig). On the left, the sample has a RIN of 10, on the right, the sample has a RIN of 5. Measurements show a 3-fold decrease of pgRNA and a 4-fold increase of HBx RNA, confirming the importance of sample quality.

In an amplitude-based multiplex assay, targets can be detected with single dye probes used at different final concentrations. This strategy described in Whale *et al.* [50]. allows the formation of distinct droplet amplitude clusters within the same fluorescence channel allowing the measurement of multiple targets per reaction and a greater separation between signals coming from probes with same dye. In our case, 4 targets are being quantified in a tetraplex reaction. HEX-probes 1 and 3 are used at 250 and 500nM respectively, and FAM-probes 2 and 4 are used at 250 and 500nM respectively [50]. With these experimental conditions, probe 1 hybridization to its target will give the equivalent of 1 HEX signal while probe 3 hybridization will generate the equivalent of 2 HEX signals [50]. Similarly, probe 2 hybridization will give 1 FAM signal while probe 4 hybridization will give 2 FAM signals. These settings offer a clearer separation of clouds and facilitate the detection of unwanted/misplaced signals (Figs 1C and 2A) due to degradation/shearing of RNAs. As example, a 3’ degraded PreS1 RNA losing the binding regions for Probe 1 and 2 will appear in the “2 HEX” quadrant instead of contaminating HBx measurements in “1 HEX” quadrant. Finally, these different relative concentrations will also allow better detection of splice variants in HBV expressing cells.

A variety of experimental settings were used for the development of ddOTs. RNAs are extracted from 4 x 10^5^ cells using RNeasy Plus Mini Kit from QIAGEN with precautions to avoid RNA shearing. RT-ddPCR is then performed using a two-step approach. cDNAs are first synthetized using Superscript IV enzyme and then used as template for droplet generation and measurements using Bio-Rad Multiplex ddPCR kit. We do not recommend the use of the Bio-Rad One-Step ddPCR kit as our results unveiled that the RT has limited ability to amplify simultaneously multiple fragments inside the same droplet.

ddPCR was performed using ddPCR Multiplex Supermix according to the manufacturer’s protocol (Bio-Rad). Controls containing DNase-treated samples without RT were systematically ran in parallel to confirm effectiveness of DNase degradation. Briefly, 5µl of diluted cDNA were mixed with 5µL 4 × ddPCR Multiplex Supermix, 1,8 µM of primers, 250 or 500 nM of each probe, 0.27µL of DTT 300mM, in a total reaction volume of 20 μL. The mixture was then partitioned into 20,000 droplets using the Automated Droplet Generator. PCR amplification was performed in a C1000 Touch thermal cycler (Bio-Rad, Hercules, CA, USA) with the following amplification program: 5 min at 95 °C, 40 cycles of denaturation for 30 s at 94 °C and annealing for 60 s at 59 °C (ramping rate set to 2 °C/s), final incubation step for 10 min at 98 °C, and kept at 4°C until fluorescence measurement. Fluorescence was measured on QX200 or QX600 Droplet Digital PCR Systems. Measurements were treated using QX Manager software.

### ddPCR data analysis

After fluorescence measurements in a QX200 (or QX600) Droplet Reader (Bio-Rad), the data were analyzed using QX Manager Software 2.3 Standard Edition (Bio-Rad), which automatically calculated absolute sample concentration. Fluorescence amplitude threshold to distinguish positive from negative droplets was based on amplification of negative controls (water, no reverse transcriptase, and non-HBV infected samples). Although not required, quantification of Rhot2 expression may be used for normalization in cases where the number of replicates is low or sample quality is variable.

### DNA extraction and cccDNA quantification

For cccDNA extraction and quantification, cells were lysed in soft lysis buffer (100mM Tris-HCl pH 8.0, 0.375% NP40) and nuclei were pelleted (1min, 14000rpm) to remove the cytoplasmic fraction. The DNA was then extracted using the DNeasy Blood and Tissue kit (QIAGEN) as per manufacturer’s instructions up to the elution where the DNA was eluted in 155μL of AE buffer. To increase the specificity of detection of the cccDNA a fraction of the samples was digested using 20U of Plasmid Safe ATP-Dependent DNase (Epicentre) at 37°C for 1h, another fraction of the samples was mock digested without the enzymes to quantify total viral DNA and cellular DNA. All samples were purified by phenol/chloroform DNA extraction before using cccDNA specific primers for qPCR quantification. The qPCR amplifications were done using the LightCycler 480 SYBR Green I system (Roche).

### Western Blot

HepG2-NTCP cells were washed with PBS and harvested in ice cold RIPA buffer supplemented with protease inhibitors (cOmplete EDTA-free, Roche) and briefly sonicated for solubilization of chromatin-associated proteins (Bioruptor Pico; 30 s ON/ 30 s OFF, 2 cycles), followed by centrifugation at 16,000 × g for 10 min at 4°C. An equal amount of total protein for each sample was run in an SDS-PAGE Mini-PROTEAN TGX (4–15%) gel (Bio-Rad). Western blot analysis was performed using anti-SMC6 (Santa Cruz: sc-365742), and anti-α-tubulin (Sigma‒Aldrich: T5168) antibodies.

## Supporting information

S1 Fig ddOTs quantification of HBV RNAs of different quality. HepG2-NTCP cells were infected with wt HBV at a MOI of 100 vge/cells and RNA was extracted 3 days post infection. Sample was split and one half was treated to induce degradation (temperature and vortex). (A) Bioanalyzer measurement of the RNA Integrity Number of non-treated sample which has a RIN of 10 (B) Bioanalyzer measurement of RNA Integrity Number for treated sample which has a RIN of 5. (C) Comparison of HBV RNA levels measurement of the two samples using ddOTs.
(TIF)

S2 Fig Multiplexed ddPCR quantification of individual synthetic DNA fragments covering the 4 HBV ORFs. Indicated PCR-amplified and purified HBV DNA fragments were quantified individually by ddOTs. (A) 2D ddPCR plot of synthetic HBx DNA fragment quantification on QX Manager Software. (B) 2D ddPCR plot of synthetic PreS2/2 DNA fragment quantification on QX Manager Software. (C) 2D ddPCR plot of synthetic PreS1 DNA fragment quantification on QX Manager Software. (D) 2D ddPCR plot of synthetic preC/pregenomic DNA fragment quantification on QX Manager Software. X-axis represents HEX signal amplitude, Y-axis represents FAM signal amplitude.
(TIF)

S3 Fig Assessment of ddOTS linearity and sensitivity. Correlation between known input concentrations and multiplexed ddPCR measurement of PCR-amplified and purified pregenomic DNA fragment from 1 to 1000 copies/µL.
(TIF)

S4 Fig Quantification of HBV RNAs levels in HepAD38 cells after Doxycycline removal. HBV RNAs expression in HepAD38 cells has been quantified by ddOTs over 96-hours time-course experiment following Doxycycline (Dox) removal. HepAD38 cells grown in presence of Dox (0.3µg/mL) for 4 weeks were plated in media without or with Dox for 96 h. Total RNA was extracted at different time points after plating and quantified by ddOTs. 0h corresponds to the time when Dox treatment was stopped. (A) Representative 2-D ddPCR plot showing the detection of each HBV RNAs in non-treated HepAD38 cells 72h after Dox removal. (B) Representative 2-D ddPCR plot showing the detection of HBV RNAs in HepAD38 cells treated with Doxycycline at time point 72h. (C) Quantification of the indicated HBV RNAs level at the indicated time after plating using ddOTs. Results are expressed as the number of copies of indicated RNA/ng of total RNA. Mean ± SEM of 3 experiments is shown.
(TIF)

S5 Fig HBV RNA levels quantification in HepAD38 cells transfected with siRNAs targeting HBx regions. HepAD38 cells were transfected with two independent siRNA targeting regions in HBx ORF that overlaps with all the HBV RNAs or with a control siRNA. 72 hours after transfection, HBV RNA levels were quantified by ddOTs. Results are expressed as the number of copies of indicated RNA/ng of total RNA in HepAD38 cells transfected with the siRNAs HBx or with siControl. Error bars represent the SEM of three independent experiments.
(TIF)

S6 Fig Assessment of ddOTs specificity for HBV targets. (A) HepG2-NTCP cells were infected with WT or X - HBV at a MOI of 100 vge/cells and nuclear DNA was collected 6 dpi. Copies number of cccDNA was quantified by qPCR. cccDNA level in HBV WT infected cells was set to 1. Error bars represent the SD of three independent experiments. (B) Total RNA was extracted from non-infected HepG2-NTCP cells and analyzed either by ddOTs (left panel) or by ddPCR for Rhot2 expression. Representative 2-D ddPCR plots for ddOTs quantification (left) and Rhot2 mRNA quantification (right) are shown. (C) HepG2-NTCP cells or PHHs were infected with wild-type HBV (WT) at an MOI of 100 vp/cell. Total RNA was collected at 6 dpi for HepG2-NTCP cells and 5 dpi for PHHs, and HBV RNAs were quantified by ddOTs under conditions in which the reverse transcriptase (RT) enzyme was omitted. Representative 2-D ddPCR plots of ddOTs quantification performed on wt HBV-infected HepG2-NTCP cells (left) and PHHs (right) in the absence of RT are shown.
(TIF)

S7 Fig Analysis of HBV RNAs kinetic in HepG2-NTCP cells using ddOTs. (A) HepG2-NTCP cells were infected with WT or X - HBV at a MOI of 100 MOI vp/cells. Nuclear DNA was collected 48h after infection and cccDNA was quantified by qPCR. cccDNA level in HBV WT infected cell was set to 1. Error bars represent the SD of three independent experiments. (B) HepG2-NTCP cells were incubated with HBV WT at a MOI of 100 MOI vp/cells for 2h without spinoculation. Total RNA was extracted at the indicated time after infection and HBV RNAs were quantified by ddOTs. Absolute quantification of the 4 main HBV RNAs and Sp1 in cells infected with WT (left). Results are expressed as number of copies per ng of total RNA. Error bars represent the SEM of three independent experiments. (C) HepG2-NTCP cells were transfected with siRNAs targeting SMC6 and infected with X - HBV at a MOI of 100 MOI vp/cells. SMC6 protein expression at different time after infection was analyzed by Western blot. Tubulin was used as loading control.
(TIF)

S8 Fig Analysis of viremia and intrahepatic HBV RNA levels in liver-humanized mice infected with HBV alone or co-infected with HBV and HDV. (A) Sera were collected at 8 weeks post-infection (wpi) from FRG liver-humanized mice (HuHep) infected either with HBV alone or with HBV and HDV. HBV and HDV viremia were analyzed by qPCR and RT-qPCR, respectively. (B) and (C) Total RNA was extracted at 8 wpi from FRG liver-humanized mice infected with HBV alone or co-infected with HBV and HDV. (B) Total HBV RNA levels were quantified using ddOTs (left panel) or RT-qPCR (right panel) and normalized to human GUS (hGUS) mRNA expression. Results are expressed as the number of RNA molecules normalized to hGUS and relative to the mean value of the HBV mono-infected group, which was set at 100%. (C) HBV pgRNA s(HBV pgRNA + HBV Sp1 RNA) were quantified using ddOTs (left panel) or RT-qPCR (right panel) and normalized to hGUS mRNA expression. Results are expressed as the number of RNA molecules normalized to hGUS and relative to the mean value of the HBV mono-infected group, which was set at 100%.
(TIF)

S9 Fig Alignment of ddOTs primers and probes with different HBV genotypes. Sequences of each set of primers and probe are shown along with the corresponding consensus sequences for HBV genotypes A to H, including genotype subtypes D1 to D4. Consensus sequences were retrieved from the Hepatitis B Virus Database. The HepAD38 reference sequences are also shown.
(TIF)

S1 Raw Image Full western for Fig S7C. (TIF)

S1 Raw data Data set for Fig 2; Fig 3; Fig.4; Fig 5; Fig 6; Fig 7; S3 Fig; S4 Fig; S5 Fig; S6 Fig, S7 Fig; S8 Fig. (XLSX)
