# Supplementary material for: ddOTs: A multiplexed quantitative ddPCR approach for resolving overlapping hepatitis B virus transcripts to decipher cccDNA-driven transcription
Source: PLoS Pathog. 2026 Jul 30;22(7):e1014464. doi: 10.1371/journal.ppat.1014464 (PMC13450838; doi:10.1371/journal.ppat.1014464)
Supplement: Supplementary file 1 [file ppat.1014464.s013.pdf]

Reviewer #1

Minor Issues:

1. The term "spanning" is used to describe primer locations, for example, in lines 135–136, the authors state that "Primers PN8, PN9, and Probe 2 (primers/probe set 2) were used for the amplification and detection of a region spanning the PreS2/S ORF." However, the two primers only amplify a region of less than 200 bp within PreS2/S, which does not truly span the ORF.

We thank the reviewer for this helpful comment. We agree that the term "spanning" was inappropriate, as the amplified fragment does not cover the entire targeted region (PreS2/S ORF, Prec/pg ORF...). We have corrected the wording accordingly. The sentences now in Material and Methods section, paragraph Primers and probe design ,reads: " Primers PN6, PN7 and Probe 1 (referred as primers/probe set 1, Table 1) were used for the amplification and detection of a region within the *HBx ORF* . Primers PN8, PN9 and Probe 2 were used for the amplification and detection of a region within the PreS2/S ORF. Primers PN10, PN11 and Probe 3 (primers/probe set 3) were used for the amplification and quantification a region within the PreS1 ORF. Primers PN12, PN13 and Probe 4 (primers/probe set 4) were used for the amplification and quantification a region within the preC/pgRNA ORF."

2. The claim that doubling the concentration of probes 3 and 4 (to 500 nM) enhances fluorescence intensity is questionable. The 3 FAM or 3 HEX mainly derives from the increased binding sites. Have the authors compared results obtained without doubling the probe concentration? Excessive probe amounts may increase background or non-specific signals rather than improving assay performance.

We thank the reviewer for this comment. We agree that simply increasing the probe concentration is not intended to enhance fluorescence of individual probe–target complexes. Our rationale follows the amplitude-based multiplexing strategy described by Whale et al. (Biomol Detect Quantif. 2016;10:15–22). In this approach, using different final probe concentrations (e.g., 50% vs. 100% of the standard concentration) allows the formation of distinct droplet amplitude clusters within the same fluorescence channel.

Therefore, the increased concentrations of probes 3 and 4 were not used to improve assay sensitivity or signal intensity, but rather to deliberately generate a higher-amplitude population, enabling clear differentiation of targets within the same fluorophore channel. This strategy also allows, for example, the separation of the PreS2/S cluster (probes 1 and 2, generating 1 HEX and 1 FAM signal) from the cluster corresponding to pSP7, pSP8, pSP9, pSP10, and pSP11 splice variants (probes 1 and 4, generating 1 HEX and 2 FAM signals). Without increasing probe concentration, both target groups would instead produce overlapping signals with 1 FAM and 1 HEX signals.

To further demonstrate that doubling probe concentration does not increase background or non-specific signals, we now provide a supplementary figure (S6B Fig) showing the 2D ddPCR plots obtained using either HBV primers and probes (ddOTs) or Rhot2 primers as a positive control, performed on RNA extracted from non-infected HepG2-NTCP cells.

To address the reviewer's concern, we have also clarified this point in the manuscript and now specify that probe concentration adjustment was performed to generate distinct amplitude clusters for multiplexing, as recommended by Whale et al. (Biomol Detect Quantif. 2016;10:15–22). In the Materials and Methods section, paragraph "ddOTs," we now state: "This strategy, described in Whale et al. (Biomol Detect Quantif. 2016;10:15–22), allows the formation of distinct

droplet-amplitude clusters within the same fluorescence channel, enabling the measurement of multiple targets within a single reaction."

3. PreC/pgRNA can be more directly and accurately quantified without duplexing if the primers and probe target the transcription start region. It may therefore be more appropriate for the ddOT approach to focus on the remaining RNA species.

We appreciate the reviewer's helpful comment. We agree that PreC/pgRNA can be quantified in a single RT-qPCR or ddPCR reaction by targeting the 5' region of pgRNA. However, this approach does not necessarily improve accuracy. When reverse transcription relies for example on random primers, any transcript retaining the 5' region, including degraded RNA fragments and several spliced variants, may be converted and amplified, potentially inflating pgRNA estimates and preventing discrimination between full-length and truncated species. The multiplex design allows differentiation between complete pgRNA and other transcripts sharing the same 5' region but differing in overall structure. For these reasons, the duplex format is better aligned with the aims of the ddOTs assay and with the complexity of HBV RNA species.

A central objective of the ddOTs assay is to quantify multiple HBV RNA species within the same reaction. Performing separate reactions would introduce additional variability (pipetting, droplet generation, partition number fluctuations). The multiplex configuration ensures that all targets are measured under identical conditions, providing more robust internal comparisons.

Finally, while we were testing the different set of primers on DNA, we verified that quantifying DNA fragment corresponding to the pgRNA by ddPCR with only the 5' primers (PN 12 and PN 11) and probe 4, or with the 4 primers and 4 probes gave the same quantification, validating the approach (Figure 1 below).

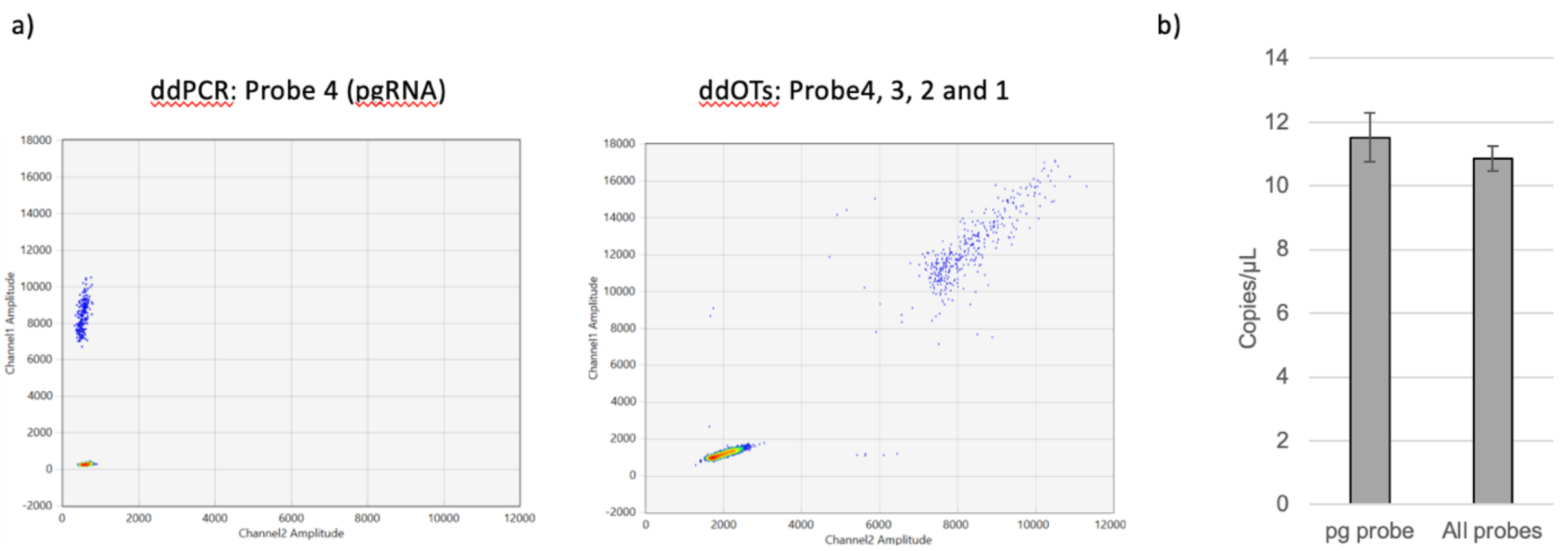

Figure 1: Quantification of DNA fragments corresponding to the pgORF using ddPCR with 5' primers alone or ddOTs. DNA was used at a concentration of 10 molecules/µl and ddPCR was performed using either PN 12 and PN11 and probe 4 or using the mix of the 4 set of primers and the 4 probes. a) 2D ddPCR plot of synthetic DNA fragment quantification on QX Manager Software. X-axis represents HEX signal amplitude; Y-axis represents FAM signal amplitude. b) Quantification by ddPCR or multiplexed ddPCR (ddOTs) of the HBV pgDNA fragment. Errors bars represent SD of 2 independent experiments.

4. The study lacks evaluation using in vivo infected samples that support robust replication, which would likely present greater complexity and provide a more realistic assessment of assay performance.

We acknowledge the reviewer's comment and used the human liver chimeric FRG mouse model (HuHep mice) infected with HBV alone or co-infected with HBV and HDV to validate our assay. We were able to identify the major HBV RNA transcripts and confirmed that HDV co-infection decreases HBV gene expression, as previously described. Importantly, these experiments were performed using frozen liver samples, and RNA was extracted using a different extraction kit, demonstrating that ddOTs can be successfully applied to frozen in vivo tissue samples and remains compatible with RNAs prepared using different extraction procedures. These data are now presented in Fig 7 and S8 Fig in the manuscript , and the manuscript text has been modified accordingly. We also added a new section entitled: "Studying HBV RNA expression in HBV-infected human liver chimeric mice".

Reviewer #2:

1.Fig. 1, pgRNA is not always having an intact 3' end, it has been reported that the 3' end of hepadnaviral encapsidated pgRNA is underrepresented due to host ribonuclease digestion after nucleocapsid formation and viral RNase H digestion during reverse transcription (PMID: 14963114; 33079954; 37754759). Therefore, the ddOTs assay may underestimate the quantity of total pgRNA. The authors may examine how their ddOTs assay will work with the isolated encapsidated pgRNA from induced HepAD38 cells and compare the pgRNA quantity in total RNA samples from HepAD38 cells with and without CAM-E treatment.

We thank the reviewer for this comment. In our experimental setup, we used for the reverse transcription step a reverse primer located at positions 1726–1746, just upstream of the 3' DR1. Based on published data (Shen et al., 2023 *PLoS Pathog*; Shen et al., 2020 *PLoS Pathog*; Ostrow & Loeb, 2004 *J. Virol.*), ddOTs therefore detect the majority of cytoplasmic pgRNA species (Shen et al., Plos Path. 2020, Fig. 7.A). Of note, pgRNA that is actively engaged in reverse transcription, and thus progressively degraded by RNase H of the viral polymerase, may not be fully quantified.

As with any RT-qPCR–based method, this approach measures steady-state levels of HBV RNA and may underestimate ongoing transcription or degradation. We also tested ddOTs using oligo(dT). HBV RNA levels were similar to those obtained with the HBx RT primer, although slightly reduced, likely due to the described 3' deletions. Depending on the objective, oligo(dT) may therefore be used to exclude 3'-trimmed encapsidated pgRNA, whereas the HBx primer allows detection of both intact and some trimmed forms.

We thank the reviewer for this important comment regarding the analysis and quantification of encapsidated HBV RNAs. Importantly, the precise identity of HBV RNA species present within capsids remains debated. Several studies have suggested that the major encapsidated species correspond to spliced variants and 3' truncated pgRNAs, while others have also reported the presence of pgRNA and HBx-derived RNAs.

Following the reviewer's suggestion, we performed additional experiments using HepAD38 cells treated or not with CAM-E for 5 days. We quantified HBV RNAs in total cellular RNA extracts and in isolated cytoplasmic capsids obtained by HBc immunoprecipitation.

As shown in Figure 2 (below), panels a (2D representation) and b (mean of 5 experiments), the four major HBV RNAs (HBx, PreS2/S, PreS1, and preC/pgRNA), as well as Sp1, were detected in HepAD38 cells regardless of CAM-E treatment. Moreover, we observed a slight increase in pgRNA species upon CAM-E treatment.

When analyzing encapsidated RNAs from mock-treated HepAD38 cells, we detected encapsidated pgRNA, Sp1, and HBV RNA species that give the same signal as PreS1 and PreS2/S respectively. We also detected substantial amounts of HBx RNAs. Upon CAM-E treatment, encapsidated pgRNA and Sp1 were strongly reduced, together with HBV RNAs species giving the same signal as PreS1 and PreS2/S. HBx RNA species were also decreased, although to a lesser extent, possibly reflecting their relatively high abundance in capsids even in untreated cells.

Overall, these ddOTs data are consistent with previously published studies showing that CAM-E treatment inhibits HBV RNA encapsidation. However, these experiments also raise several important points.

First, ddOTs analysis may underestimate encapsidated pgRNA levels. Indeed, Shen and colleagues demonstrated that encapsidated pgRNA displays heterogeneous 3' ends due to progressive 3' degradation, with termini frequently located upstream of the region recognized by the RT primer used in our ddOTs assay (Shen et al., PLoS Pathogens 2020; J. Virol. 2023).

Second, we detected HBV RNA species amplified by the PreS2/S and PreS1 primer/probe sets. Similar RNA species of approximately 2.4 kb and 2.1 kb were previously detected by Northern blotting (Shen et al., PLoS Pathogens 2020; J. Virol. 2023). The authors proposed that such shorter RNAs correspond to spliced variants identified by 3' RACE analysis. Interestingly, several splice variants (Sp2, Sp3, Sp4, Sp5, Sp10, Sp16, and Sp17) could theoretically be detected using the PreS2/S primer/probe set, while Sp8 could be detected using the PreS1 set. Shen and colleagues specifically identified Sp8 and Sp19 spliced variants in HepAD38 cells (supplementary Figure 3 in Shen et al., PLoS Pathogens 2020). In addition, Sp3 has been described as a common RNA species in encapsidated HBV capsids (Lam et al., Antimicrobial Agents and Chemotherapy 2017).

Finally, one particularly intriguing observation is the detection of HBx RNA species within capsids. This phenomenon has been reported by several groups using approaches such as 5' RACE and RNA-seq (Stadelmayer et al., J. Hepatol. 2020; Niu et al., PLoS One 2017; Paturel et al., JHEP Reports 2024). The mechanism by which HBx RNAs, which lack the canonical 5' encapsidation signal, become encapsidated remains unclear. Stadelmayer et al. detected both capped and uncapped HBx RNAs of variable lengths and proposed that these RNAs might arise from endolytic cleavage of pgRNA by the RNase H domain of the viral polymerase rather than direct transcription from cccDNA. Alternatively, pgRNA degradation by cellular 5'→3' exoribonucleases such as XRN1 during encapsidation or within capsids could generate the uncapped HBx RNAs observed by Stademayer et al. In support of this possibility, Wada and colleagues recently demonstrated that HBV pgRNA and envelope RNAs are targets of nonsense-mediated RNA decay (Wada et al., iScience 2025). 5' degradation mechanisms could potentially generate not only HBx RNAs of variable lengths, but also RNA fragments overlapping the PreS2/S and PreS1 regions.

We would however like to emphasize that the primary objective of this study was to develop ddOTs as a tool to quantify the different HBV RNA species in order to analyze the transcriptional activity

of the various HBV promoters, as well as the biology of HBV RNAs in the cytoplasm (stability, transport, etc.). Nevertheless, we believe that the reviewers' suggestions to apply ddOTs to additional questions, such as the characterization and quantification of encapsidated HBV RNAs, further illustrate the strong potential of this approach for studying different aspects of HBV replication and HBV RNA biology. We agree that such applications would be highly valuable; however, they would likely require additional methodological adaptations of ddOTs depending on the specific biological question being addressed and therefore remain beyond the scope of the present study. In particular, at this stage, we cannot definitively conclude that the HBV RNA species detected by ddOTs within capsids correspond exclusively to spliced variants.

a)

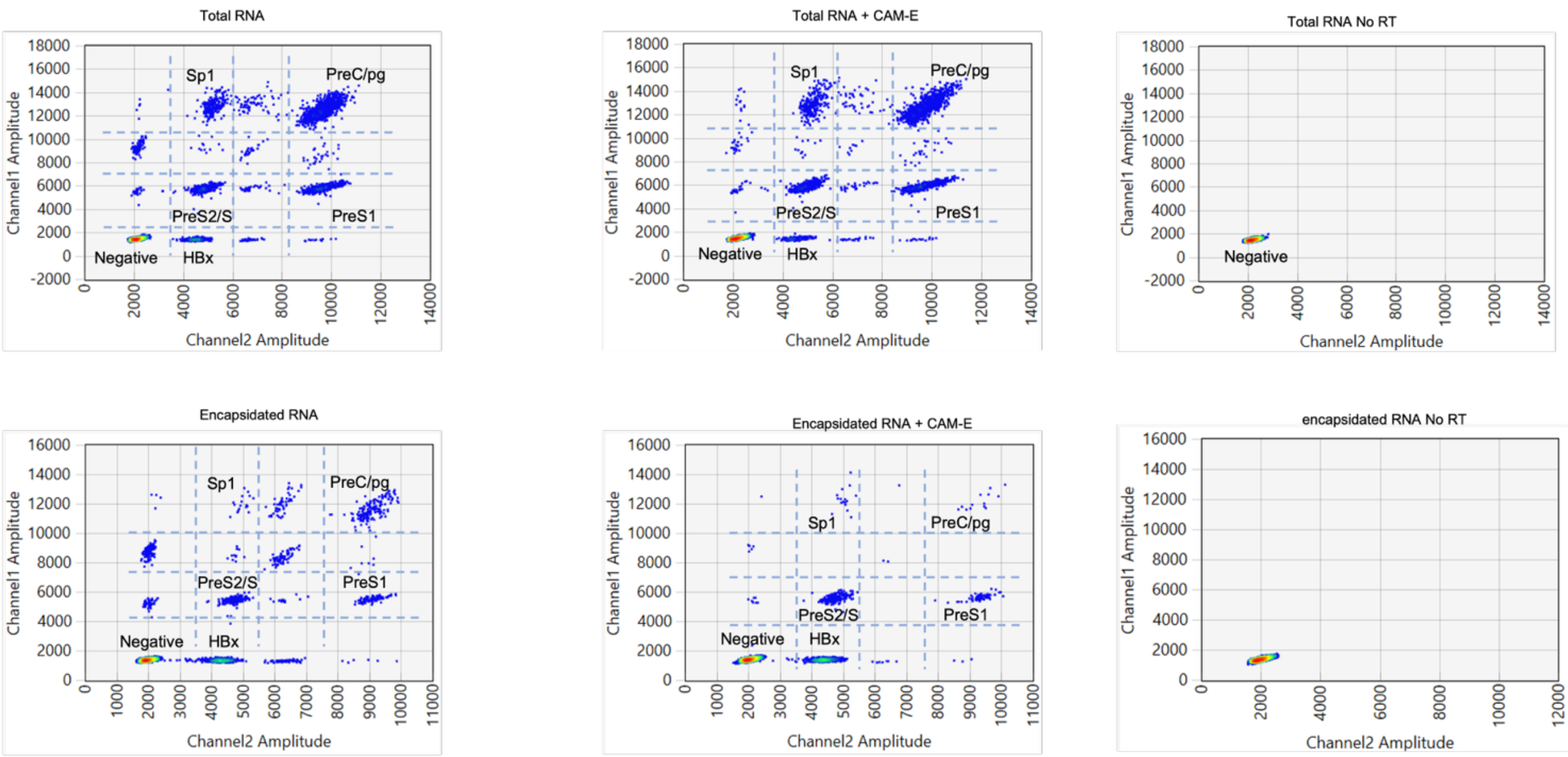

b)

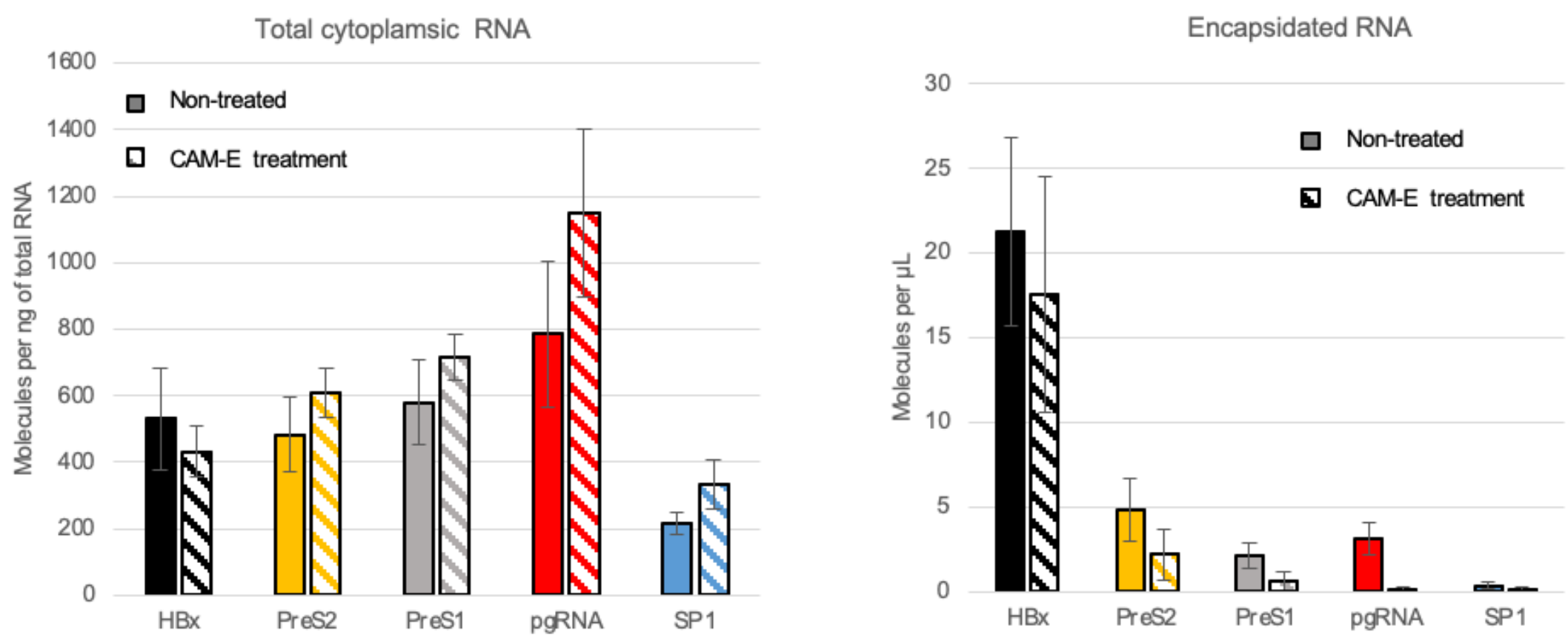

Figure 2: **Quantification of encapsidated HBV RNAs levels in HepAD38**
HepAD38 cells were cultured in the presence or absence of CAM-E (JnJ827, Lahlali et al., AAC 2018) at a concentration of 1 µM for 5 days. Total RNA was extracted as described in the Materials and Methods section (RNA extraction paragraph). For cytoplasmic encapsidated HBV RNAs, capsids were prepared as described in Shen et al., *Journal of Virology* (2023, Vol. 97), using an anti-HBc antibody (MA1-7607). Following immunoprecipitation, encapsidated RNAs were extracted using RLT buffer and processed using the same protocol as for total RNA extraction.
(a) Two-dimensional ddPCR plots showing detection of HBV RNAs in untreated or CAM-E-treated HepAD38 cells, in total RNA extracts or in isolated capsids. (b) Quantification of the indicated HBV RNA levels using ddOTs. Results are expressed as copies of the indicated RNA per ng of total RNA for total RNA extracts, or as molecules per µL for encapsidated RNAs. Data represent mean ± SD from 5 experiments.

2. Fig.3, were the HepAD38 cells cultured in tet-free medium during cell maintenance and passage? If yes, this is not a common practice with the tet-off HBV stable cell lines. Ideally, the authors should assess the kinetics of HBV RNA transcription in HepAD38 cells by withdrawing tet from the culture medium for a time-course experiment and subsequently adding tet back for a second time-course analysis. It would also be interesting to see what HBV RNA can be detected from the supernatant of HepAD38 cells by ddOTs.

We thank the reviewer for this relevant comment. We performed a time-course experiment using HepAD38 cells initially maintained in the presence of tetracycline, followed by tetracycline withdrawal.

Our results show that HBV RNA expression starts to increase 48 hours after tetracycline removal, with a more pronounced effect observed for pgRNA levels. This is consistent with the fact that pgRNA transcription is directly controlled by the tetracycline-regulated CMV promoter in HepAD38 cells. The data are now shown in S4 Fig . The main text has been corrected accordingly.

The questions regarding (1) the analysis of encapsidated HBV RNA in the context of CAM-E treatment and (2) here the detection of HBV RNA released into the supernatant are closely related and address the same underlying issue, while we agree that HBV RNAs present in the supernatant can be associated either with viral capsids or, in part, with extracellular vesicles such as exosomes. However as recently reported (Paturel et al.2024), the majority of extracellular HBV RNAs in tissue culture model are found in encapsidated and enveloped forms rather than in bona fide exosomes.

Therefore, while we agree with the reviewer that the development of methods enabling the precise discrimination and quantification of encapsidated HBV RNA species would be highly valuable, we believe that additional methodological adaptations of ddOTs will be required for this specific application.

3. Fig.4, an HBx siRNA could be included as a control.

We acknowledge the reviewer's comment and performed additional experiments using two siRNAs targeting the HBx region, which overlaps with all HBV transcripts and may therefore lead to a reduction in all HBV RNA species. As expected, silencing this region resulted in a decrease in all HBV RNA species detected by ddOTs.

These data are now presented in S5 Figure , and the manuscript text has been modified accordingly in the section entitled "Analysis of HBx RNA expression levels in HepAD38 cells by ddOTs" (line 191).

4. Fig.6, cccDNA quantification data should be used for viral RNA normalization between wt and X- virus infection. The X- virus data can be further strengthened by HBx transcomplementation, followed by ddOTs. Was the X- virus produced from 1.3HBVΔX cell line by transfecting HBx plasmid? Regarding the putative NTCP-independent entry of HBx mRNA, was it caused by spinoculation? The authors may re-examine the early phase HBx mRNA level during X- virus infection without the spinoculation step.

cccDNA quantification data should be used for viral RNA normalization between wt and X- virus infection.

We thank the reviewer for this comment, which also addresses a minor point raised by reviewer 3. We agree with the reviewer's comment and we routinely control the infection by assessing cccDNA level which may serve if needed for normalization of RNA levels. cccDNA levels related to experiments shown in Fig 5 are shown in S6A Figure and cccDNA levels related to experiments shown in Fig 6 are shown in S7A Figure .

The X- virus data can be further strengthened by HBx transcomplementation, followed by ddOTs.

We thank the reviewer for this insightful suggestion. We agree that HBx transcomplementation would be a valuable approach to further strengthen the X^{-} virus data. However, to maintain consistency with the experimental design presented in Figure 6 and to enable the use of the four sets of primers and probes, we opted to restore HBV X^{-} expression by silencing SMC6 rather than performing HBx transcomplementation. Indeed, transcomplementation with HBx would have required the exclusion of the HBx-specific primer and probe, thereby limiting our ability to comprehensively assess all viral transcripts.

As now shown in Fig 6C, silencing SMC6 resulted in an increase in all HBV RNA species at 24 hours post-infection, reaching levels comparable to those observed with wild-type virus infection. In contrast, HBV RNA levels in control siRNA-treated cells remained low, similar to those observed in HBV X^{-} infection.

Was the X- virus produced from 1.3HBVΔX cell line by transfecting HBx plasmid?

No, the virus was produced directedly from 1.3HBVΔX cell. Of note, HBx expression is required only for the transcription of the viral episome not for the transcription of the integrated HBV genome (Van Breugel et al., Hepatology 2012).

Regarding the putative NTCP-independent entry of HBx mRNA, was it caused by spinoculation? The authors may re-examine the early phase HBx mRNA level during X- virus infection without the spinoculation step.

We thank the reviewer for this important comment and for raising the possibility that the observed NTCP-independent entry of HBx mRNA could be influenced by spinoculation. To address this point, we performed infection experiments without the spinoculation step.

Overall, the kinetics of HBV RNA expression for wild-type (WT) virus were comparable to those observed with spinoculation. In WT infection, HBx mRNA levels began to increase between 8 and 12 hours post-infection, whereas pgRNA, preS2, and preS1 RNA levels increased later, after 12 hours, with a marked rise at 24 hours post-infection consistent with the results obtained with spinoculation.

Importantly, we were still able to detect HBx RNA at early time points during WT infection. However, these levels remained extremely low (below 0.05 molecules/ng RNA), similar to those observed with spinoculation. It remains unclear whether such low levels reflect a biologically relevant function (e.g., serving as a template for HBx translation) or rather residual input RNA that enters the cell and is subsequently degraded. These data are now presented in S7B Figure , and the manuscript text has been modified accordingly in the section entitled :" Analysis of dynamics of HBV RNA expression in early stages of infection by ddOTs" (line 284).

While the reviewer's question is highly relevant, addressing the precise origin and functional significance of these early HBx RNA would require further dedicated investigations. This is beyond the scope of the present study, which primarily aims to validate a new technique for the specific quantification of individual HBV RNA species, and which will be instrumental for addressing such mechanistic questions in future studies.

Minor Issues:

1. Fig.1, the ddOTs assay still cannot distinguish precore mRNA from pgRNA, while the reported 5' RACE method can. Does the short 5' extension on pC mRNA versus pgRNAallow a pC mRNA-specific ddPCR in ddOTs?

We thank the reviewer for this comment. While it is currently not possible with our setup to distinguish preC RNA from pgRNA, this differentiation is nonetheless technically feasible. To achieve this, one could design a forward primer targeting the short 5' extension specific to preC mRNA and a reverse primer targeting a region in pgRNA. Because our RT step uses a primer located downstream of the 3' region shared by all HBV RNAs and duplicated at the 5' end of both preC RNA and pgRNA, the resulting cDNAs would retain a unique 5' region that could be exploited to discriminate between these two transcripts.

Although such a multiplexed ddPCR assay could theoretically be performed using only two fluorophores, the use of additional fluorochromes would further improve cluster identification and separation. However, despite the availability of suitable fluorophores, the current Bio-Rad QX Manager software does not allow the simultaneous quantification of molecules labeled with more

than three distinct fluorochromes. Once this software limitation is resolved, the development of new primer/probe sets labeled with different fluorophores will enable clearer separation and more accurate discrimination of these transcripts.

2. Line 42, remove “of”.

fixed.

3. Correct “HepaD38” to “HepAD38” throughout the manuscript.

Fixed

4. Lines 317-318, please further explain why and how other spliced forms of HBV RNA can be detected by ddOTs.

As shown in Figure 3 (below), in addition to the main HBV transcripts, several additional signals “clouds” (A, B, C, D, E, F, G, H, I, and J) are detected using ddOTS. Importantly, these clouds represent only a minor fraction of the total HBV RNA population (approximately 5–6%). In this experiment, the measured concentrations for the major transcripts were: pgRNA, 116 copies/µL; Sp1, 14.2 copies/µL; PreS1, 124 copies/µL; PreS2/S, 80.3 copies/µL; and HBx, 22.1 copies/µL. In contrast, the combined abundance of clouds A+C+D+F+E+G+H+I+J reached only 18.3 copies/µL.

Based on their position in the 2D plot, we know which probes hybridized to each reverse-transcribed DNA molecule, allowing us to infer the likely identity of the corresponding spliced variants. For example, cloud A corresponds to molecules hybridizing with probe 2 and probe 4, which is compatible with the spliced variants TCONS_00049 and TCONS_00067 described by Lim *et al.* after analysis of 501 RNA-seq libraries (Lim *et al.*, *Microbial Genomics* 2021; 7:000492, DOI: 10.1099/mgen.0.000492) (Figure 4, below). However, we note that molecules in cloud A could also arise from partial degradation of Sp1 RNA.

Similarly, molecules in cloud F match the expected hybridization pattern for spliced variants TCONS_00006, TCONS_00018, or TCONS_00072, also reported by Lim *et al.* (Lim *et al.*, *Microbial Genomics* 2021; 7:000492) (see Fig. 3 and 4). Because these molecules retain the 5' probe 4 and the 3' probe 1, they necessarily correspond to spliced species.

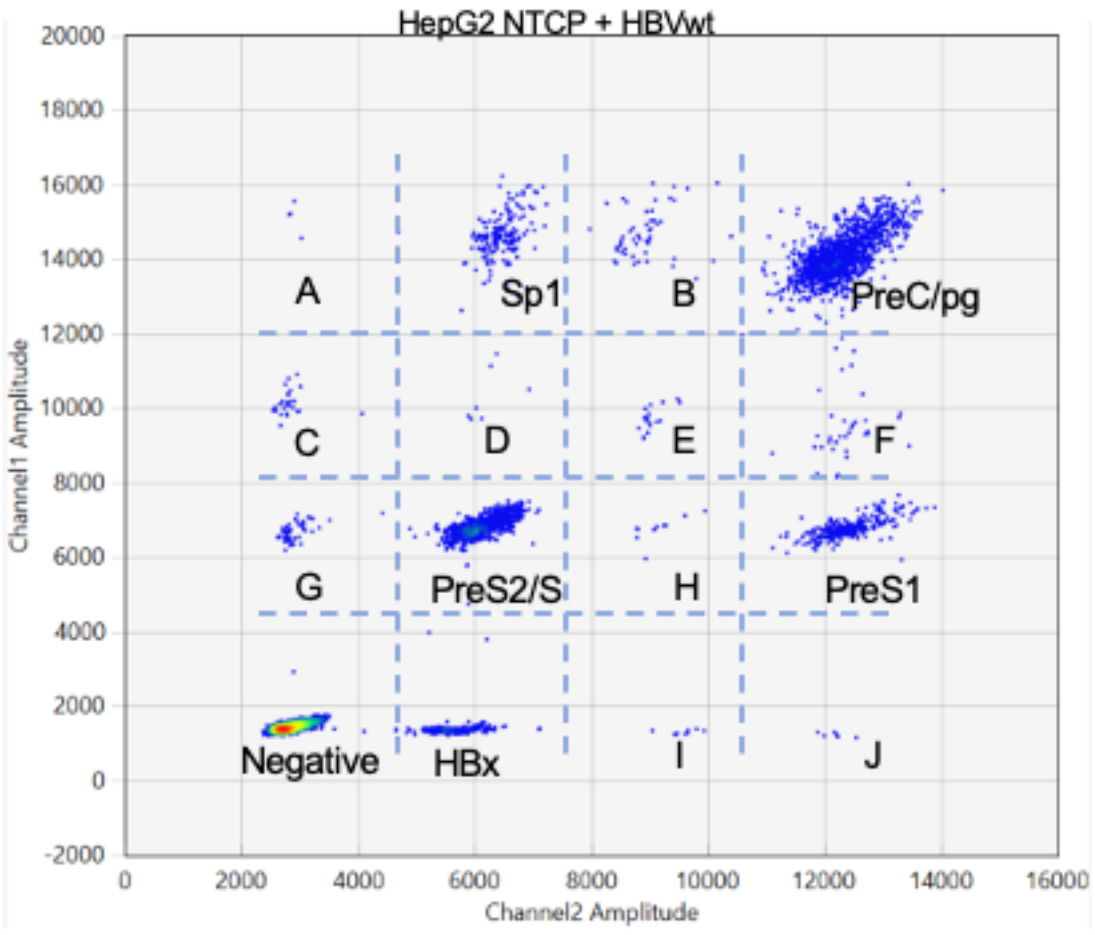

A : TCONS_00049, TCONS_00067
B : TCONS_00057
C : TCONS_00055, TCONS_00056
D : pSP7, pSP8, pSP9, pSP10, pSP11
E :
F : TCONS_00006, TCONS_00018, TCONS_00072
G : TCONS_00052, TCONS_00059
H : TCONS_00061, TCONS_00068
I :
J : TCONS_00025, TCONS_00026, TCONS_00043

Figure 3: HepG2-NTCP cells were infected with WT HBV at a MOI of 100 vge/cells. Total RNA was collected 6 dpi and HBV RNAs were quantified by ddOTs. 2D ddPCR plot of HBV RNAs analysis in HepG2-NTCP cells infected with HBV WT is shown.

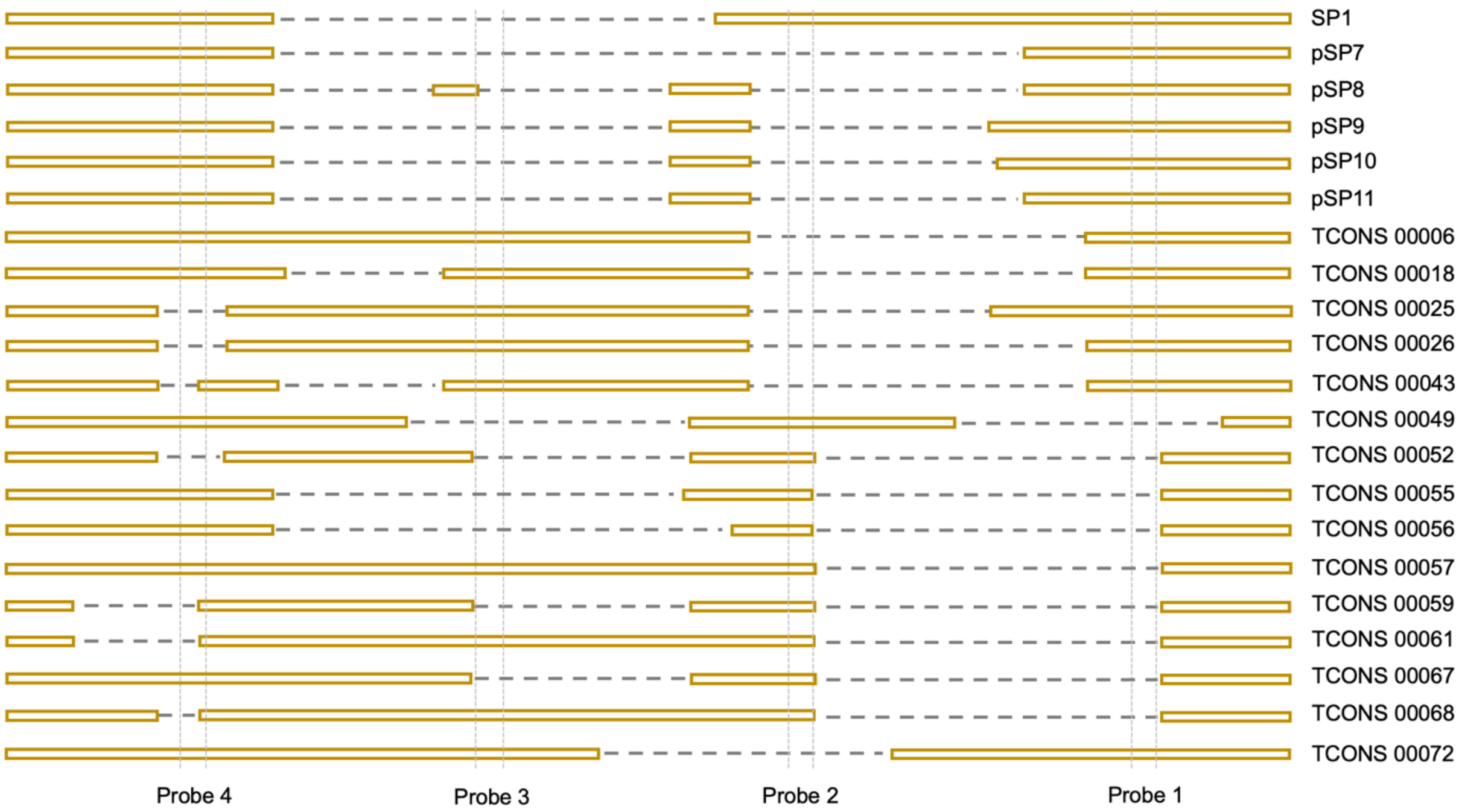

Figure 4: Spliced variants adapted from Lim and collaborators (Lim et al., Microbial Genomics 2021;7, DOI 10.1099/mgen.0.000492). The localization of the probes used in our study is shown below the spliced HBV RNAs.

Finally, as we noted in the manuscript, "the development of probes with additional fluorochromes will facilitate the identification of spliced variants. At present, limitations of the Bio-Rad QX Manager software prevent quantification of molecules simultaneously bound by more than three fluorochromes." For instance, the design of new probes positioned between probes 2 and 3 should allow discrimination between the Sp1 and Sp9 spliced variants.

Reviewer #3:

DNA contamination control : The authors should clarify how they ensured that the PCR signal does not originate from residual DNA molecules (e.g., rcDNA or cccDNA). This needs to be demonstrated experimentally. A simple control would be to perform the PCR without the RT step or in the presence of a reverse transcriptase inhibitor.

We thank the reviewer for this important comment and for raising this critical point regarding potential DNA contamination in the assay. We fully agree that it is essential to ensure that the PCR signal does not originate from residual DNA molecules such as rcDNA or cccDNA, and we appreciate the opportunity to clarify this aspect.
As stated in the Materials and Methods section, "Controls containing DNase-treated samples without RT were systematically run in parallel to verify the efficacy of DNase digestion."

Importantly, we have now included S6C Figure, which presents representative 2-D ddPCR plots showing that ddPCR performed on DNase-treated samples without RT from infected PHH or HepG2-NTCP cells yielded almost exclusively negative droplets, thereby confirming the absence of detectable residual DNA.

Exosomal RNA secretion

HBV RNAs are known to be secreted in exosomes, particularly during long-term HBV infection. It would be interesting to investigate whether and to what extent viral RNAs are found in the culture supernatant or viral input compared to intracellular fractions.

We thank the reviewer for this interesting suggestion regarding the analysis of HBV RNAs in exosomes.

We would however like to emphasize that the primary objective of this study was to develop ddOTs as a tool to quantify the different HBV RNA species in order to analyze the transcriptional activity of the various HBV promoters, as well as the biology of HBV RNAs in the cytoplasm (stability, transport, etc.). Nevertheless, we believe that the reviewers' suggestions to apply ddOTs to additional questions, such as the quantification of HBV RNAs in the culture supernatant further illustrate the strong potential of this approach for studying different aspects of HBV replication and HBV RNA biology. We agree that such applications would be highly valuable; however, they would likely require additional methodological adaptations of ddOTs.

Exosome purification requires dedicated and standardized methodologies that are not currently established in our laboratory. In addition, although our RNA extraction procedure is fully compatible with high-quality RNA (RIN > 8, and often up to RIN 10), additional upstream fractionation steps required for exosome isolation (including prolonged centrifugation and multiple handling steps) may affect RNA integrity and would likely require careful optimization of the workflow.

We therefore consider that the analysis of exosome-associated HBV RNAs, while of potential interest, would require the development and standardization of dedicated protocols, and is beyond the scope of the present work. We believe that such approaches should be optimized on a case-by-case basis depending on the specific biological questions addressed.

Genotype sequence variability and primer binding

A crucial question is whether the protocol can also be applied to other HBV genotypes, such as HBV genotype A, B, or C. HBV genotype D exhibits numerous sequence variations. This should be experimentally demonstrated or, at the very least, discussed in detail. The authors should include a supplementary figure showing the consensus sequences of genotype D and other genotypes aligned to the primer and probe binding sites. This would help to assess which HBV genotypes are compatible with the current assay design.

We thank the reviewer for his suggestion and we added a figure showing the alignment with HBV genotype D subtypes 1,2,3 and 4 and the HBV genotype A,B, C, D, E, F (S9 Fig).
The effects of mismatches on PCR amplification have been studied for both primers and probes and data have suggested that mismatch at the 3'-end of primer had more detrimental effect on real time PCR than nucleotide changes located at the 5-end of the primer. The type of

mismatches: purine-purine, pyrimidine-pyrimidine, purine-pyrimidine also impact differently the amplification with the purine-purine mismatch being the most detrimental. Finally, less of 3 mismatches in one primer may have no major impact on amplification. Mismatch in the probe binding region is believed to be more detrimental if located in the center of the sequence (Howson et al., Pathogens, 2020 vol 9, 303; Stadhouders et al., J. Mol. Diagn., 2010, vol 12, 109 ; Lefever et al., Clinical Chemistry , 2013, vol 59, 1740; SÜß et al. J. Microbiol. Methods 2009, 76, 316). Interestingly we have the opportunity to sequence the HBV genome used to engineered the HepAD38 cells (provided by Pr Christophe Seeger) and the analysis of the sequence showed changes similar to the genotype D3 (HepAD38 sequences are now shown in Supplemental Figure 9). In accordance with published data regarding primers/probes mismatches consequence on PCR amplification we observed that our primers/probe sets are able to efficiently amplify the HBV genome in HepAD38 cells as well HBV X- genome similar to the consensus subgenotype D sequence.
The detection of the other HBV genotypes, because of the higher number of mismatches in the probe or in the primer's sequences, will require the development of specific set of primers or as suggested in the discussion, the development of degenerated probes and primers as developed to quantify HIV reservoir using cross-subtype multiplex ddPCR (Cassidy et al., IScience Volume 25, Issue 1, 103615).
This point is now discussed in the second paragraph of the Discussion, and HBV genotypes are presented in S9 Figure .

Figure labeling and RNA populations

In Figures 4 and 5, distinct populations are visible (e.g., Channel 1 Amp 6000 or 10000; Channel 2 Amp 3000). The Authors should explain why these clear populations appear and what their origin is. Moreover, the authors should standardize the figure labeling and clearly indicate which populations correspond to which RNA transcripts (Sp1, PreC/pg, PreS2/S, PreS1, HBx) throughout the manuscript.

In Figures 4 and 5, distinct populations are visible (e.g., Channel 1 Amp 6000 or 10000; Channel 2 Amp 3000). The Authors should explain why these clear populations appear and what their origin is.

We thank the reviewer for this comment, which also addresses point 4 in minor comments raised by reviewer 1. As shown in Figure 3 (below), in addition to the main HBV transcripts, several additional signals "clouds" (A, B, C, D, E, F, G, H, I, and J) are detected using ddOTS. Importantly, these clouds represent only a minor fraction of the total HBV RNA population (approximately 5–6%). In this experiment, the measured concentrations for the major transcripts were: pgRNA, 116 copies/µL; Sp1, 14.2 copies/µL; PreS1, 124 copies/µL; PreS2/S, 80.3 copies/µL; and HBx, 22.1 copies/µL. In contrast, the combined abundance of clouds A+C+D+F+E+G+H+I+J reached only 18.3 copies/µL.

Based on their position in the 2D plot, we know which probes hybridized to each reverse-transcribed DNA molecule, allowing us to infer the likely identity of the corresponding spliced variants. The population that appears in G (e.g., Channel 1 Amp 6000 and Channel 2 Amp 3000) corresponds to molecules hybridizing with probe 2 which is compatible with the spliced variants TCONS_00052, or TCONS_00059, also reported by Lim et al. (Lim et al., Microbial Genomics 2021; 7:000492) (see Fig. 3 and 4). We note that molecules in cloud G could also arise from partial degradation of PreS2/2. Again, cloud G represents less than 1% of the total positive droplets.

The population that appears in C (e.g., Channel 1 Amp 10000 and Channel 2 Amp 3000) corresponds to molecules hybridizing with probe 4 only, which is compatible with the spliced variants TCONS_00055 and TCONS_00056 described by Lim et al.(Lim et al., Microbial Genomics 2021; 7:000492). However, we note that molecules in cloud C could also arise from partial degradation of pgRNA or Sp1 RNA molecules. To note, cloud C represents less than 1% of the total positive droplets.

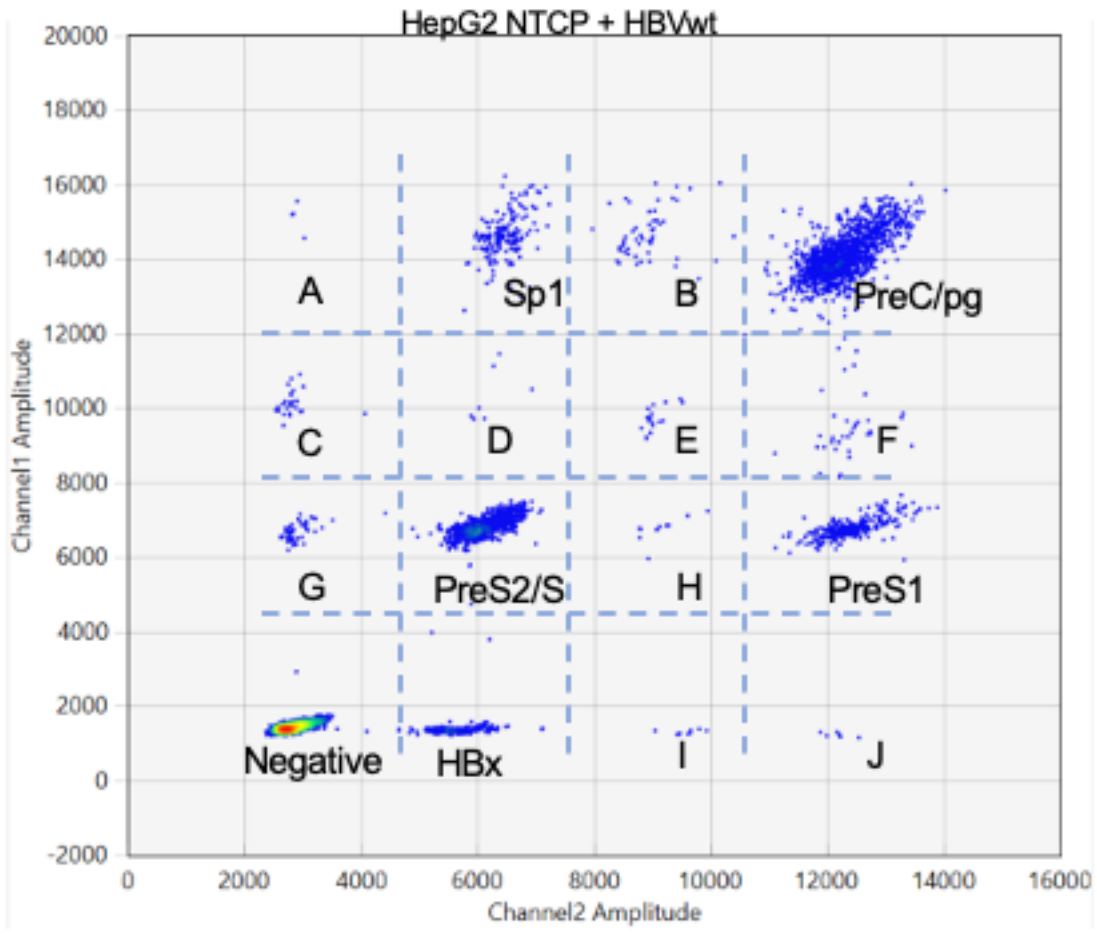

A : TCONS_00049, TCONS_00067
B : TCONS_00057
C : TCONS_00055, TCONS_00056
D : pSP7, pSP8, pSP9, pSP10, pSP11
E :
F : TCONS_00006, TCONS_00018, TCONS_00072
G : TCONS_00052, TCONS_00059
H : TCONS_00061, TCONS_00068
I :
J : TCONS_00025, TCONS_00026, TCONS_00043

Figure 3: HepG2-NTCP cells were infected with WT HBV at a MOI of 100 vge/cells. Total RNA was collected 6 dpi and HBV RNAs were quantified by ddOTs. 2D ddPCR plot of HBV RNAs analysis in HepG2-NTCP cells infected with HBV wt is shown.

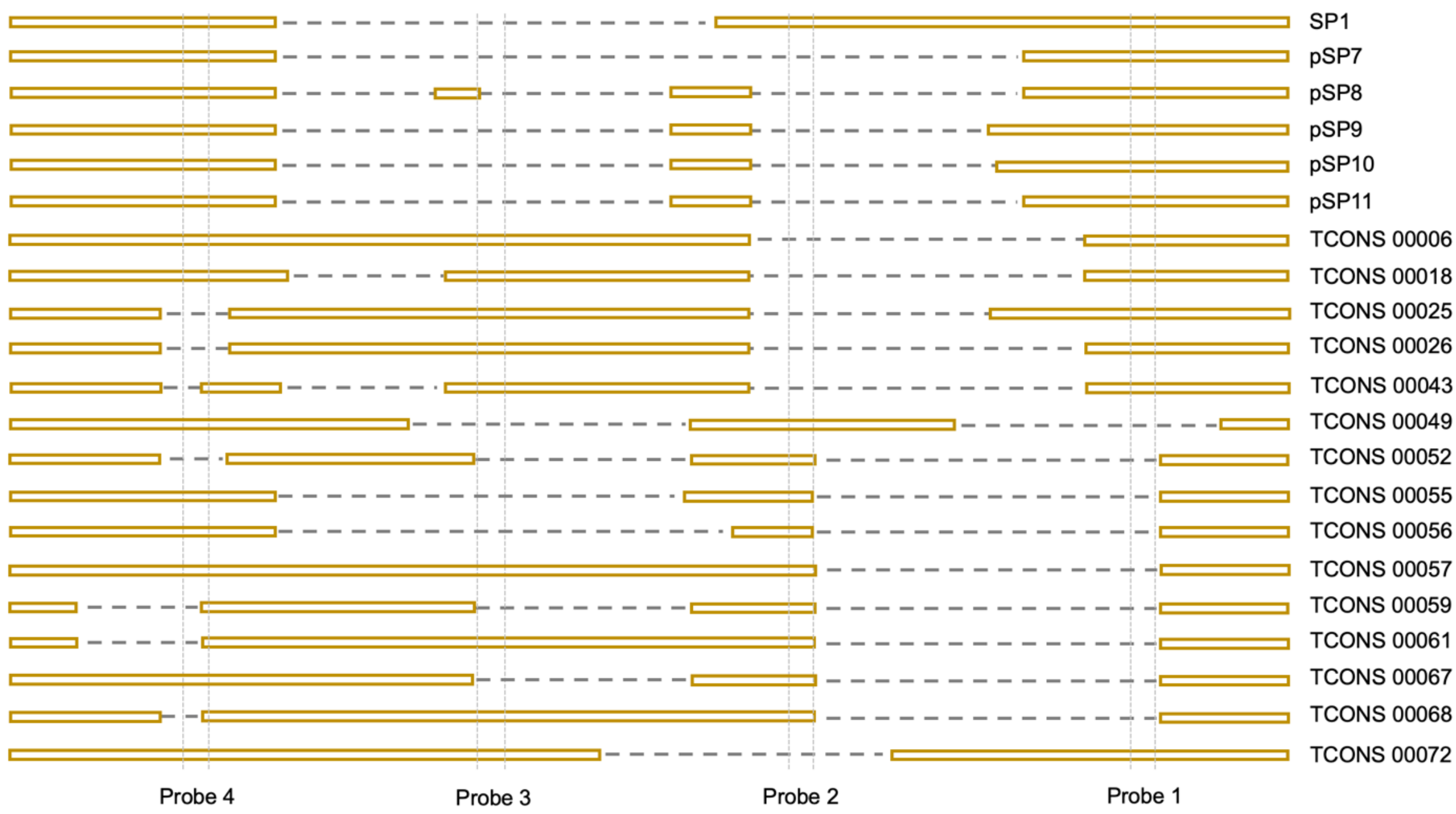

Figure 4: Spliced variants adapted from Lim and collaborators (Lim et al., Microbial Genomics 2021;7, DOI 10.1099/mgen.0.000492). The localization of the probes used in our study is shown below the spliced HBV RNAs.

Moreover, the authors should standardize the figure labeling and clearly indicate which populations correspond to which RNA transcripts (Sp1, PreC/pg, PreS2/S, PreS1, HBx) throughout the manuscript.

We thank the reviewer for this suggestion, and we have now labeled all the 2D plots accordingly.

Elongated droplet populationsThe authors should briefly explain why some droplet populations appear stretched. Does this indicate that multiple RNA molecules are present in a single droplet, or that amplification does not proceed equally across all probes?

We thank the reviewer for this relevant comment. On the 2D-plot, each positive droplet gives a fluorescence signal accordingly to the dye associated. This signal can vary from droplet to droplet (up to 20 000 droplets per reaction) due to differences in droplet size or temperature. As an example, probe 1 hydrolysis will give a signal expected around 6000 on Channel2 but can vary from 5000 to 7000 on x-axis due to the high number of positive droplets and variability associated. In the same way, probe 2 hydrolysis gives a signal around 6000, but can vary between 5000 and 7000 on y-axis. When only 1 probe binds to the target molecule, this heterogeneity only appears on a 1-dimensional axis. If two different dye probes bind to the target molecule, the population will seem stretched on the X-axis and on the Y-axis. This phenomenon does not imply a less efficient amplification but will result in a population appearing stretched for

molecules bound by multiples probes. pgRNA being bound by 4 probes, the population will appear more stretched than preS2/S population for example.

Minor Issues:

The summary claims that the method can be used to study "how this DNA produces the different viral RNAs, how it is regulated, and how the virus persists." While the assay can indeed quantify the different RNA transcripts, answering these broader biological questions requires additional experiments and complementary approaches. The authors should adopt a more cautious wording here.

We thank the reviewer for this comment and have modified the sentence accordingly. In the Author Summary section (lines 48–51), we now write: "This method allows accurate and detailed quantification of viral RNA transcripts, providing a useful additional tool to examine aspects of viral gene expression. By enabling such analyses even from rare or small biological samples, our approach can facilitate future studies aimed at understanding viral persistence and identifying potential antiviral targets."

Methods: medium composition

The methods section states that 1% P/S was used; the concentration of the P/S stock solution should be specified. Additionally, the description of the Williams E medium used for HepG2 H1.3dX cells lacks information about additives. The same applies to the Williams E production medium.

We thank the reviewer for this comment. We used a stock solution of Penicillin/Streptomycin containing 10,000 units of penicillin and 10 mg/mL of streptomycin. We have now specified the stock solution concentration in the *Materials and Methods* section, under *Cell culture, HBV production, and infection*. We have also described the culture conditions used for maintaining hepAD38 and HepG2 H1.3Δx cells, as well as the culture conditions used for virus production.

Formatting issues and Typos

We first thanks the reviewer for his comments and we fixed all the mentioned typos.

Line 113: missing space between for and 1h30min.

fixed

Line 159: should read 10^5 instead of 105.

fixed

Line 479: HepAD38 should be spelled correctly.

Fixed

Line 474: should read models (plural)

Fixed

Supplementary Figure 2: should read copies.

Fixed

Viral input normalization: It is difficult to directly compare wtHBV and HBV X- infections at the RNA level unless identical viral inputs are confirmed. Although the authors state that viral particles were compared using antibody precipitation, this does not necessarily guarantee identical rcDNA-to-dslDNA ratios, which could affect infection efficiency.

We thank the reviewer for this comment, which also addresses point 4 raised by reviewer 2. As suggested, we assessed HBV cccDNA levels after infecting HepG2-NTCP cells with either HBV wt or HBV X^- virus. Our results show that infection at an MOI of 100 vge/cell leads to comparable amounts of cccDNA for both viruses. These data have now been included as Supplemental Figure. cccDNA levels related to experiments shown in Fig 5 are shown in S6A Figure and cccDNA levels related to experiments shown in Fig 6 are shown in S7A Figure . The text in the Results section has been modified accordingly in the Results section (lines 214-215 and 254) .

Viral input units. The authors use inconsistent units to describe viral input ("vp/cell" vs. "vge/cell"). These should be standardized throughout the manuscript.

We acknowledge the reviewer comment and we have now harmonized the manuscript by using vge/cells

Infection blocking controls
While the authors used MyrB to block infection, which is appropriate, they also note NTCP-independent entry. Including heparin or anti-envelope antibodies as additional controls could help confirm complete inhibition of viral attachment

We are grateful for the reviewer's suggestion to consider additional entry inhibitors such as heparin or anti-envelope antibodies. However, we believe that Myrcludex B (MyrB) alone is sufficient in our experimental context for the following reasons. MyrB is widely recognized as the most potent and specific inhibitor of HBV entry currently available. It binds directly to NTCP and blocks the critical preS1–NTCP interaction required for productive infection. Several independent studies have demonstrated that MyrB inhibits HBV and HDV entry in vitro with sub-nanomolar to even picomolar potency (IC_{50} as low as ~80 pM). In our experiments, we used MyrB at concentrations previously shown to robustly prevent infection. Under these conditions, the efficacy of MyrB has consistently been validated across multiple hepatocyte models, including primary human hepatocytes and NTCP-expressing cell lines, with minimal cytotoxicity or off-target effects. As a result, MyrB has become the standard entry inhibitor in the HBV/HDV field, and its use alone is widely accepted in peer-reviewed studies.
In the present work, we used MyrB to block HBV entry in order to challenge our ddOTs approach and evaluate whether it accurately monitors HBV RNA expression under different replicative conditions. We fully agree with the reviewer that, if our future goal is to study early infection events and determine whether early-detected RNAs originate from cccDNA transcription after its establishment or from incoming viral particles containing RNA, via NTCP-dependent or NTCP-independent pathways, then employing additional entry inhibitors and complementary approaches will indeed be necessary.
