# Supplementary material for: ddOTs: A multiplexed quantitative ddPCR approach for resolving overlapping hepatitis B virus transcripts to decipher cccDNA-driven transcription
Source: PLoS Pathog. 2026 Jul 30;22(7):e1014464. doi: 10.1371/journal.ppat.1014464 (PMC13450838; doi:10.1371/journal.ppat.1014464)
Supplement: Supplementary file 2 [file ppat.1014464.s014.docx]

Reviewer #1

None

Reviewer #2:

None

Reviewer #3:

First, the authors state in the text: “We designed four pairs of primers/probes sets targeting the 4 main HBV ORFs (HBx, PreS2/S, PreS1 and preC/pgRNA).” However, Figure 1B appears to show the HBV mRNAs rather than the ORFs. In contrast, Figure 1A shows the HBV genome and the corresponding ORFs. I therefore suggest either indicating the primer/probe positions in Figure 1A or adding the ORF annotations to Figure 1B to avoid confusion, or correct the statement in den text.

We thank the reviewer for this helpful comment. To avoid any ambiguity, we revised the text by replacing the sentence: "We designed four pairs of primers/probes sets targeting the 4 main HBV ORFs (HBx, PreS2/S, PreS1 and preC/pgRNA)." with: "We designed four pairs of primers/probes sets targeting the four main HBV RNAs (HBx, PreS2/S, PreS1 and preC/pgRNA)." in the Result section line 102.

Second, the overall figure quality is very poor, at least in the version provided for review. The numbers and individual labels are difficult to read. However, I noticed that Figure 6A appears to be missing the x-axis legend.

We thank the reviewer for pointing this out. We carefully revised the quality of all figures by improving the readability of the labels, axis annotations, tick labels, and graph resolution to enhance clarity for the readers. In addition, the missing x-axis label in Figure 6A has been added. Furthermore, we used the PLOS NAAS tool to convert all figure files into high-quality TIFF files, in accordance with the journal's recommendations.
