# Supplementary material for: ddOTs: A multiplexed quantitative ddPCR approach for resolving overlapping hepatitis B virus transcripts to decipher cccDNA-driven transcription
Source: PLoS Pathog. 2026 Jul 30;22(7):e1014464. doi: 10.1371/journal.ppat.1014464 (PMC13450838; doi:10.1371/journal.ppat.1014464)
Supplement: Peer review history

# Peer review: Decision Letter 0

**DOI:** 10.1371/journal.ppat.1014464.r001

**Authors:** Blossom Damania, Jianming Hu

12 Nov 2025

-->PPATHOGENS-D-25-02516

ddOTs: a multiplexed quantitative ddPCR approach for resolving overlapping hepatitis B virus transcripts to decipher cccDNA-driven transcription

PLOS Pathogens

Dear Dr. Neuveut,

Thank you for submitting your manuscript to PLOS Pathogens. After careful consideration, we feel that it has merit but does not fully meet PLOS Pathogens's publication criteria as it currently stands. In particular, all three reviewers raised significant conceptual and technical issues that should be addressed. Therefore, we invite you to submit a revised version of the manuscript that addresses the points raised during the review process.

Please submit your revised manuscript by Jan 11 2026 11:59PM. If you will need more time than this to complete your revisions, please reply to this message or contact the journal office at plospathogens@plos.org. When you're ready to submit your revision, log on to https://www.editorialmanager.com/ppathogens/ and select the 'Submissions Needing Revision' folder to locate your manuscript file.

Please include the following items when submitting your revised manuscript:

* A rebuttal letter that responds to each point raised by the editor and reviewer(s). You should upload this letter as a separate file labeled 'Response to Reviewers'. This file does not need to include responses to any formatting updates and technical items listed in the 'Journal Requirements' section below.

* A marked-up copy of your manuscript that highlights changes made to the original version. You should upload this as a separate file labeled 'Revised Manuscript with Track Changes'.

* An unmarked version of your revised paper without tracked changes. You should upload this as a separate file labeled 'Manuscript'.

If you would like to make changes to your financial disclosure, competing interests statement, or data availability statement, please make these updates within the submission form at the time of resubmission. Guidelines for resubmitting your figure files are available below the reviewer comments at the end of this letter.

We look forward to receiving your revised manuscript.

Kind regards,

Jianming Hu

Academic Editor

PLOS Pathogens

Blossom Damania

Section Editor

PLOS Pathogens

Sumita Bhaduri-McIntosh

Editor-in-Chief

PLOS Pathogens

orcid.org/0000-0003-2946-9497

Michael Malim

-->Editor-in-Chief

PLOS Pathogens

orcid.org/0000-0002-7699-2064

**Journal Requirements:**

If the reviewer comments include a recommendation to cite specific previously published works, please review and evaluate these publications to determine whether they are relevant and should be cited. There is no requirement to cite these works unless the editor has indicated otherwise.

1) Please ensure that the CRediT author contributions listed for every co-author are completed accurately and in full.

At this stage, the following Authors/Authors require contributions: Nazim Sarica, Irene Meki, Oceane Lopez, Basile Jay, Gael Petitjean, and Christine Neuveut. Please ensure that the full contributions of each author are acknowledged in the "Add/Edit/Remove Authors" section of our submission form.

The list of CRediT author contributions may be found here: https://journals.plos.org/plospathogens/s/authorship#loc-author-contributions

2) Please upload all main figures as separate Figure files in .tif or .eps format. For more information about how to convert and format your figure files please see our guidelines:

https://journals.plos.org/plospathogens/s/figures

3) We have noticed that you have uploaded Supporting Information files, but you have not included a list of legends. Please add a full list of legends for your Supporting Information files after the references list.

4) We note that your Data Availability Statement is currently as follows: "All data is included in the manuscript and/orsupporting information". Please confirm at this time whether or not your submission contains all raw data required to replicate the results of your study. Authors must share the “minimal data set” for their submission. PLOS defines the minimal data set to consist of the data required to replicate all study findings reported in the article, as well as related metadata and methods (https://journals.plos.org/plosone/s/data-availability#loc-minimal-data-set-definition).

For example, authors should submit the following data:

1) The values behind the means, standard deviations and other measures reported;

2) The values used to build graphs;

3) The points extracted from images for analysis..

Authors do not need to submit their entire data set if only a portion of the data was used in the reported study.

If your submission does not contain these data, please either upload them as Supporting Information files or deposit them to a stable, public repository and provide us with the relevant URLs, DOIs, or accession numbers. For a list of recommended repositories, please see https://journals.plos.org/plosone/s/recommended-repositories.

If there are ethical or legal restrictions on sharing a de-identified data set, please explain them in detail (e.g., data contain potentially sensitive information, data are owned by a third-party organization, etc.) and who has imposed them (e.g., an ethics committee). Please also provide contact information for a data access committee, ethics committee, or other institutional body to which data requests may be sent. If data are owned by a third party, please indicate how others may request data access.

5) Please amend your detailed Financial Disclosure statement. This is published with the article. It must therefore be completed in full sentences and contain the exact wording you wish to be published.

1) State the initials, alongside each funding source, of each author to receive each grant. For example: "This work was supported by the National Institutes of Health (####### to AM; ###### to CJ) and the National Science Foundation (###### to AM)."

2) State what role the funders took in the study. If the funders had no role in your study, please state: "The funders had no role in study design, data collection and analysis, decision to publish, or preparation of the manuscript."

3) If any authors received a salary from any of your funders, please state which authors and which funders..

If you did not receive any funding for this study, please simply state: u201cThe authors received no specific funding for this work.u201d

6) Kindly revise your competing statement in the online submission form to align with the journal's style guidelines: 'The authors declare that there are no competing interests.'

**Reviewers' Comments:**

Reviewer's Responses to Questions

**Part I - Summary**

Please use this section to discuss strengths/weaknesses of study, novelty/significance, general execution and scholarship.

Reviewer #1: This study introduces a novel approach, ddOT (droplet digital PCR for Overlapping Transcripts), for quantifying five distinct species of HBV viral RNAs. Accurately measuring each viral RNA species has long been a technical challenge, and this multiplex ddPCR method represents meaningful progress toward resolving it. However, the assay’s accuracy depends on the integrity of each RNA species. In reality, HBV RNA metabolism in infected cells is dynamic and subject to degradation—for example, pgRNA is degraded during reverse transcription. Consequently, partially degraded pgRNA molecules may not be detected by this method, potentially leading to an underestimation of RNA levels.

Reviewer #2: In this manuscript, the authors report the development of a multiplexed ddPCR method, called ddOTs, to specifically quantify the 5 major HBV transcripts with overlapping 3’ ends, including the 3.5kb pC/pgRNA, 2.4kb L mRNA, 2.1kb M/S mRNA, 0.7kb HBx mRNA, and the 2.2kb pC/pgRNA major splicing product SP1. The rationale behind the assay design is that each viral mRNA possesses a distinct 5′ extension length, allowing the use of specific combinations of PCR amplicons and fluorescent probes in a ddPCR assay. The authors conducted validating experiments of the ddOTs assay in multiple settings with synthetic samples and cell culture samples. They also used the assay to monitor the kinetics and dynamics of HBV transcripts during HBV infection of cell culture models in the presence of absence of HBx expression. Overall, this study demonstrates innovation in ddOTs assay development, and the application of ddOTs to analyze cccDNA-dependent transcripts generated several interesting results, albeit largely confirmatory of prior observations. The author may consider the following comments to further strengthen the manuscript.

Reviewer #3: Sarica et al. have developed and established a multiplex quantitative assay based on digital droplet PCR (ddPCR) to distinguish and quantify different HBV RNA transcripts within a single assay. The assay was validated using both synthetic DNA oligos and HepAD38 cells that stably express HBV. In a final step, the authors applied the assay to compare RNA transcript profiles between wild-type HBV (wtHBV) and HBV X-deficient infections. The authors present an elegant experimental design and methodology to analyze and quantify the ratios of various HBV RNA transcripts. This approach shows clear potential for studying how different factors influence HBV transcriptional regulation

The manuscript presents a technically robust and conceptually innovative ddPCR-based assay with significant potential for studying HBV transcriptional regulation. However, addressing the points below would considerably strengthen the rigor and clarity of the work.

**********

**Part II – Major Issues: Key Experiments Required for Acceptance**

Please use this section to detail the key new experiments or modifications of existing experiments that should be absolutely required to validate study conclusions.

Generally, there should be no more than 3 such required experiments or major modifications for a "Major Revision" recommendation. If more than 3 experiments are necessary to validate the study conclusions, then you are encouraged to recommend "Reject".

Reviewer #1: None

Reviewer #2: 1. Fig. 1, pgRNA is not always having an intact 3’ end, it has been reported that the 3’ end of hepadnaviral encapsidated pgRNA is underrepresented due to host ribonuclease digestion after nucleocapsid formation and viral RNase H digestion during reverse transcription (PMID: 14963114; 33079954; 37754759). Therefore, the ddOTs assay may underestimate the quantity of total pgRNA. The authors may examine how their ddOTs assay will work with the isolated encapsidated pgRNA from induced HepAD38 cells and compare the pgRNA quantity in total RNA samples from HepAD38 cells with and without CAM-E treatment.

2. Fig.3, were the HepAD38 cells cultured in tet-free medium during cell maintenance and passage? If yes, this is not a common practice with the tet-off HBV stable cell lines. Ideally, the authors should assess the kinetics of HBV RNA transcription in HepAD38 cells by withdrawing tet from the culture medium for a time-course experiment and subsequently adding tet back for a second time-course analysis. It would also be interesting to see what HBV RNA can be detected from the supernatant of HepAD38 cells by ddOTs.

3. Fig.4, an HBx siRNA could be included as a control.

4. Fig.6, cccDNA quantification data should be used for viral RNA normalization between wt and X- virus infection. The X- virus data can be further strengthened by HBx transcomplementation, followed by ddOTs. Was the X- virus produced from 1.3HBVΔX cell line by transfecting HBx plasmid? Regarding the putative NTCP-independent entry of HBx mRNA, was it caused by spinoculation? The authors may re-examine the early phase HBx mRNA level during X- virus infection without the spinoculation step.

Reviewer #3: DNA contamination control

The authors should clarify how they ensured that the PCR signal does not originate from residual DNA molecules (e.g., rcDNA or cccDNA). This needs to be demonstrated experimentally. A simple control would be to perform the PCR without the RT step or in the presence of a reverse transcriptase inhibitor.

Exosomal RNA secretion

HBV RNAs are known to be secreted in exosomes, particularly during long-term HBV infection. It would be interesting to investigate whether and to what extent viral RNAs are found in the culture supernatant or viral input compared to intracellular fractions.

Genotype sequence variability and primer binding

A crucial question is whether the protocol can also be applied to other HBV genotypes, such as HBV genotype A, B, or C. HBV genotype D exhibits numerous sequence variations. This should be experimentally demonstrated or, at the very least, discussed in detail. The authors should include a supplementary figure showing the consensus sequences of genotype D and other genotypes aligned to the primer and probe binding sites. This would help to assess which HBV genotypes are compatible with the current assay design.

Figure labeling and RNA populations

In Figures 4 and 5, distinct populations are visible (e.g., Channel 1 Amp 6000 or 10000; Channel 2 Amp 3000). The Authors should explain why these clear populations appear and what their origin is. Moreover, the authors should standardize the figure labeling and clearly indicate which populations correspond to which RNA transcripts (Sp1, PreC/pg, PreS2/S, PreS1, HBx) throughout the manuscript.

Elongated droplet populations

The authors should briefly explain why some droplet populations appear stretched. Does this indicate that multiple RNA molecules are present in a single droplet, or that amplification does not proceed equally across all probes?

**********

**Part III – Minor Issues: Editorial and Data Presentation Modifications**

Please use this section for editorial suggestions as well as relatively minor modifications of existing data that would enhance clarity.

Reviewer #1: 1. The term “spanning” is used to describe primer locations, for example, in lines 135–136, the authors state that “Primers PN8, PN9, and Probe 2 (primers/probe set 2) were used for the amplification and detection of a region spanning the PreS2/S ORF.” However, the two primers only amplify a region of less than 200 bp within PreS2/S, which does not truly span the ORF.

2. The claim that doubling the concentration of probes 3 and 4 (to 500 nM) enhances fluorescence intensity is questionable. The 3 FAM or 3 HEX mainly derives from the increased binding sites. Have the authors compared results obtained without doubling the probe concentration? Excessive probe amounts may increase background or non-specific signals rather than improving assay performance.

3. PreC/pgRNA can be more directly and accurately quantified without duplexing if the primers and probe target the transcription start region. It may therefore be more appropriate for the ddOT approach to focus on the remaining RNA species.

4. The study lacks evaluation using in vivo infected samples that support robust replication, which would likely present greater complexity and provide a more realistic assessment of assay performance.

Reviewer #2: 1. Fig.1, the ddOTs assay still cannot distinguish precore mRNA from pgRNA, while the reported 5’ RACE method can. Does the short 5’ extension on pC mRNA versus pgRNA allow a pC mRNA-specific ddPCR in ddOTs?

2. Line 42, remove “of”.

3. Correct “HepaD38” to “HepAD38” throughout the manuscript.

4. Lines 317-318, please further explain why and how other spliced forms of HBV RNA can be detected by ddOTs.

Reviewer #3: Author summary wording

The summary claims that the method can be used to study “how this DNA produces the different viral RNAs, how it is regulated, and how the virus persists.” While the assay can indeed quantify the different RNA transcripts, answering these broader biological questions requires additional experiments and complementary approaches. The authors should adopt a more cautious wording here.

Methods: medium composition

The methods section states that 1% P/S was used; the concentration of the P/S stock solution should be specified. Additionally, the description of the Williams E medium used for HepG2 H1.3dX cells lacks information about additives. The same applies to the Williams E production medium.

Formatting issues and Typos

Line 113: missing space between for and 1h30min.

Line 159: should read 10⁵ instead of 105.

Line 479: HepAD38 should be spelled correctly.

Line 474: should read models (plural).

Supplementary Figure 2: should read copies.

Viral input normalization

It is difficult to directly compare wtHBV and HBV X- infections at the RNA level unless identical viral inputs are confirmed. Although the authors state that viral particles were compared using antibody precipitation, this does not necessarily guarantee identical rcDNA-to-dslDNA ratios, which could affect infection efficiency.

Viral input units

The authors use inconsistent units to describe viral input (“vp/cell” vs. “vge/cell”). These should be standardized throughout the manuscript.

Infection blocking controls

While the authors used MyrB to block infection, which is appropriate, they also note NTCP-independent entry. Including heparin or anti-envelope antibodies as additional controls could help confirm complete inhibition of viral attachment.

**********

PLOS authors have the option to publish the peer review history of their article (what does this mean?). If published, this will include your full peer review and any attached files.

If you choose “no”, your identity will remain anonymous but your review may still be made public.

**Do you want your identity to be public for this peer review?**  For information about this choice, including consent withdrawal, please see our Privacy Policy.

Reviewer #1: No

Reviewer #2: No

Reviewer #3: No

[NOTE: If reviewer comments were submitted as an attachment file, they will be attached to this email and accessible via the submission site. Please log into your account, locate the manuscript record, and check for the action link "View Attachments". If this link does not appear, there are no attachment files.]

**Figure resubmission:**

-->While revising your submission, we strongly recommend that you use PLOS’s NAAS tool (https://ngplosjournals.pagemajik.ai/artanalysis) to test your figure files. NAAS can convert your figure files to the TIFF file type and meet basic requirements (such as print size, resolution), or provide you with a report on issues that do not meet our requirements and that NAAS cannot fix.

After uploading your figures to PLOS’s NAAS tool - https://ngplosjournals.pagemajik.ai/artanalysis, NAAS will process the files provided and display the results in the "Uploaded Files" section of the page as the processing is complete. If the uploaded figures meet our requirements (or NAAS is able to fix the files to meet our requirements), the figure will be marked as "fixed" above. If NAAS is unable to fix the files, a red "failed" label will appear above. When NAAS has confirmed that the figure files meet our requirements, please download the file via the download option, and include these NAAS processed figure files when submitting your revised manuscript.

**Reproducibility:**

To enhance the reproducibility of your results, we recommend that authors of applicable studies deposit laboratory protocols in protocols.io, where a protocol can be assigned its own identifier (DOI) such that it can be cited independently in the future. Additionally, PLOS ONE offers an option to publish peer-reviewed clinical study protocols. Read more information on sharing protocols at https://plos.org/protocols?utm_medium=editorial-email&utm_source=authorletters&utm_campaign=protocols

# Author comment: Author response to Decision Letter 1

**DOI:** 10.1371/journal.ppat.1014464.r002

18 May 2026

Attachment Submitted filename: Response to Reviewers.pdf

# Peer review: Decision Letter 1

**DOI:** 10.1371/journal.ppat.1014464.r003

**Authors:** Blossom Damania, Jianming Hu

1 Jul 2026

PPATHOGENS-D-25-02516R1

ddOTs: a multiplexed quantitative ddPCR approach for resolving overlapping hepatitis B virus transcripts to decipher cccDNA-driven transcription

PLOS Pathogens

Dear Dr. Neuveut,

Thank you for submitting your manuscript to PLOS Pathogens. After careful consideration, we feel that it has merit but does not fully meet PLOS Pathogens's publication criteria as it currently stands. In particular, one reviewer has some minor issues that you should address. Therefore, we invite you to submit a revised version of the manuscript that addresses those points.

Please submit your revised manuscript by Aug 30 2026 11:59PM. If you will need more time than this to complete your revisions, please reply to this message or contact the journal office at plospathogens@plos.org. When you're ready to submit your revision, log on to https://www.editorialmanager.com/ppathogens/ and select the 'Submissions Needing Revision' folder to locate your manuscript file.

Please include the following items when submitting your revised manuscript:

* A letter that responds to each point raised by the editor and reviewer(s). You should upload this letter as a separate file labeled 'Response to Reviewers'. This file does not need to include responses to any formatting updates and technical items listed in the 'Journal Requirements' section below.

* A marked-up copy of your manuscript that highlights changes made to the original version. You should upload this as a separate file labeled 'Revised Manuscript with Track Changes'.

* An unmarked version of your revised paper without tracked changes. You should upload this as a separate file labeled 'Manuscript'.

If you would like to make changes to your financial disclosure, competing interests statement, or data availability statement, please make these updates within the submission form at the time of resubmission. Guidelines for resubmitting your figure files are available below the reviewer comments at the end of this letter.

As the corresponding author, your ORCID iD is verified in the submission system and will appear in the published article. PLOS supports the use of ORCID, and we encourage all coauthors to register for an ORCID iD and use it as well. Please encourage your coauthors to verify their ORCID iD within the submission system before final acceptance, as unverified ORCID iDs will not appear in the published article. Only the individual author can complete the verification step; PLOS staff cannot verify ORCID iDs on behalf of authors.

We look forward to receiving your revised manuscript.

Kind regards,

Jianming Hu

Academic Editor

PLOS Pathogens

Blossom Damania

Section Editor

PLOS Pathogens

Sumita Bhaduri-McIntosh

Editor-in-Chief

PLOS Pathogens

orcid.org/0000-0003-2946-9497

Michael Malim

Editor-in-Chief

PLOS Pathogens

orcid.org/0000-0002-7699-2064

**Additional Editor Comments (if provided):**

**Reviewers' Comments:**

Reviewer's Responses to Questions

**Part I - Summary**

Please use this section to discuss strengths/weaknesses of study, novelty/significance, general execution and scholarship.

Reviewer #1: The authors thoroughly addressed each of all comments and performed additional experiments. The results provide additional support for the robustness of their ddOT assay. Publication of this study may facilitate studying HBV virology and evaluating antiviral responses.

Reviewer #2: The authors have addressed my comments adequately. The existence and origin of the early HBx mRNA remain unclear, but the authors have acknowledged and discussed the limitations of this finding in the revised manuscript.

Reviewer #3: The authors, Sarica et al., have addressed all points raised in my previous review and have responded to them satisfactorily. In its current form, the manuscript has improved substantially, both scientifically and linguistically.

Upon re-evaluation of the revised manuscript, I noticed two remaining minor points that the authors should correct before publication. See below.

In summary, I consider the manuscript and the novel technology described therein to represent an important contribution to the current field of research. I therefore recommend acceptance after correction of these minor points.

**********

**Part II – Major Issues: Key Experiments Required for Acceptance**

Please use this section to detail the key new experiments or modifications of existing experiments that should be absolutely required to validate study conclusions.

Generally, there should be no more than 3 such required experiments or major modifications for a "Major Revision" recommendation. If more than 3 experiments are necessary to validate the study conclusions, then you are encouraged to recommend "Reject".

Reviewer #1: None

Reviewer #2: (No Response)

Reviewer #3: (No Response)

**********

**Part III – Minor Issues: Editorial and Data Presentation Modifications**

Please use this section for editorial suggestions as well as relatively minor modifications of existing data that would enhance clarity.

Reviewer #1: None

Reviewer #2: (No Response)

Reviewer #3: First, the authors state in the text: “We designed four pairs of primers/probes sets targeting the 4 main HBV ORFs (HBx, PreS2/S, PreS1 and preC/pgRNA).” However, Figure 1B appears to show the HBV mRNAs rather than the ORFs. In contrast, Figure 1A shows the HBV genome and the corresponding ORFs. I therefore suggest either indicating the primer/probe positions in Figure 1A or adding the ORF annotations to Figure 1B to avoid confusion, or correct the statement in den text.

Second, the overall figure quality is very poor, at least in the version provided for review. The numbers and individual labels are difficult to read. However, I noticed that Figure 6A appears to be missing the x-axis legend.

**********

PLOS authors have the option to publish the peer review history of their article (what does this mean?). If published, this will include your full peer review and any attached files.

If you choose “no”, your identity will remain anonymous but your review may still be made public.

**Do you want your identity to be public for this peer review?**  For information about this choice, including consent withdrawal, please see our Privacy Policy.

Reviewer #1: No

Reviewer #2: No

Reviewer #3: **Yes:**  Ulrike Protzer

[NOTE: If reviewer comments were submitted as an attachment file, they will be attached to this email and accessible via the submission site. Please log into your account, locate the manuscript record, and check for the action link "View Attachments". If this link does not appear, there are no attachment files.]

**Figure resubmission:**

-->While revising your submission, we strongly recommend that you use PLOS’s NAAS tool (https://ngplosjournals.pagemajik.ai/artanalysis) to test your figure files. NAAS can convert your figure files to the TIFF file type and meet basic requirements (such as print size, resolution), or provide you with a report on issues that do not meet our requirements and that NAAS cannot fix.

After uploading your figures to PLOS’s NAAS tool - https://ngplosjournals.pagemajik.ai/artanalysis, NAAS will process the files provided and display the results in the "Uploaded Files" section of the page as the processing is complete. If the uploaded figures meet our requirements (or NAAS is able to fix the files to meet our requirements), the figure will be marked as "fixed" above. If NAAS is unable to fix the files, a red "failed" label will appear above. When NAAS has confirmed that the figure files meet our requirements, please download the file via the download option, and include these NAAS processed figure files when submitting your revised manuscript.

**Reproducibility:**

To enhance the reproducibility of your results, we recommend that authors of applicable studies deposit laboratory protocols in protocols.io, where a protocol can be assigned its own identifier (DOI) such that it can be cited independently in the future. Additionally, PLOS ONE offers an option to publish peer-reviewed clinical study protocols. Read more information on sharing protocols at https://plos.org/protocols?utm_medium=editorial-email&utm_source=authorletters&utm_campaign=protocols

# Author comment: Author response to Decision Letter 2

**DOI:** 10.1371/journal.ppat.1014464.r004

8 Jul 2026

Attachment Submitted filename: Response to reviewers.docx

# Editor report: Decision Letter 2

**DOI:** 10.1371/journal.ppat.1014464.r005

**Authors:** Blossom Damania, Jianming Hu

9 Jul 2026

Dear Dr Neuveut,

We are pleased to inform you that your manuscript 'ddOTs: a multiplexed quantitative ddPCR approach for resolving overlapping hepatitis B virus transcripts to decipher cccDNA-driven transcription' has been provisionally accepted for publication in PLOS Pathogens.

Before your manuscript can be formally accepted you will need to complete some formatting changes, which you will receive in a follow up email. A member of our team will be in touch with a set of requests.

Please note that your manuscript will not be scheduled for publication until you have made the required changes, so a swift response is appreciated.

IMPORTANT: The editorial review process is now complete. PLOS will only permit corrections to spelling, formatting or significant scientific errors from this point onwards. Requests for major changes, or any which affect the scientific understanding of your work, will cause delays to the publication date of your manuscript.

Should you, your institution's press office or the journal office choose to press release your paper, you will automatically be opted out of early publication. We ask that you notify us now if you or your institution is planning to press release the article. All press must be co-ordinated with PLOS.

Thank you again for supporting Open Access publishing; we are looking forward to publishing your work in PLOS Pathogens.

Best regards,

Jianming Hu

Academic Editor

PLOS Pathogens

Blossom Damania

Section Editor

PLOS Pathogens

Sumita Bhaduri-McIntosh

Editor-in-Chief

PLOS Pathogens

orcid.org/0000-0003-2946-9497

Michael Malim

Editor-in-Chief

PLOS Pathogens

orcid.org/0000-0002-7699-2064

***********************************************************

Reviewer Comments (if any, and for reference):

# Editor report: Acceptance letter

**DOI:** 10.1371/journal.ppat.1014464.r006

**Authors:** Blossom Damania, Jianming Hu

Dear Dr Neuveut,

We are delighted to inform you that your manuscript, "ddOTs: a multiplexed quantitative ddPCR approach for resolving overlapping hepatitis B virus transcripts to decipher cccDNA-driven transcription," has been formally accepted for publication in PLOS Pathogens.

We have now passed your article onto the PLOS Production Department who will complete the rest of the pre-publication process. All authors will receive a confirmation email upon publication.

The corresponding author will soon be receiving a typeset proof for review, to ensure errors have not been introduced during production. Please review the PDF proof of your manuscript carefully, as this is the last chance to correct any scientific or type-setting errors. Please note that major changes, or those which affect the scientific understanding of the work, will likely cause delays to the publication date of your manuscript. Note: Proofs for Front Matter articles (Pearls, Reviews, Opinions, etc...) are generated on a different schedule and may not be made available as quickly.

Soon after your final files are uploaded, the early version of your manuscript, if you opted to have an early version of your article, will be published online. The date of the early version will be your article's publication date. The final article will be published to the same URL, and all versions of the paper will be accessible to readers.

For Research Articles, you will receive an invoice from PLOS for your publication fee after your manuscript has reached the completed accept phase. If you receive an email requesting payment before acceptance or for any other service, this may be a phishing scheme. Learn how to identify phishing emails and protect your accounts at https://explore.plos.org/phishing.

Thank you again for supporting open-access publishing; we are looking forward to publishing your work in PLOS Pathogens.

Best regards,

Sumita Bhaduri-McIntosh

Editor-in-Chief

PLOS Pathogens

orcid.org/0000-0003-2946-9497

Michael Malim

Editor-in-Chief

PLOS Pathogens

orcid.org/0000-0002-7699-2064
